# Supplementary material for: Noise distracts foraging bats
Source: Proc Biol Sci. 2021 Feb 10;288(1944):20202689. doi: 10.1098/rspb.2020.2689 (PMC7893219; doi:10.1098/rspb.2020.2689)
Supplement: Noise distracts foraging bats - supplement [file rspb20202689supp1.pdf]

Pallids in Noise Supplement

8/9/2020

```r
##reading in video data
dat1<-read.csv("BatsInNoise_8-5.csv")
dat1$Bat<-as.factor(dat1$Bat)


###reading in audio data
dat3<-read.csv("Echolocation_BatsInNoise_8-5.csv")
dat3$Bat<-as.factor(dat3$Bat)
dat3<-droplevels(subset(dat3, Attempt!="0"))
```

Attempt models

The following models test the effect of noise on bats' willingness to make a foraging attempt

```r
#reording levels of Treatment
dat1$Treatment<-factor(dat1$Treatment, levels=c("Ambient", "Overlapping", "Non-overlapping"))

###Model testing the effect of prey reward on trial outcome###
Attempt_mod1<-glm(Attempt~Treatment*MW_absent, family=binomial(link="logit"), data=dat1)
summary(Attempt_mod1)
```

```
##
## Call:
## glm(formula = Attempt ~ Treatment * MW_absent, family = binomial(link = "logit"),
##     data = dat1)
##
## Deviance Residuals:
##     Min       1Q   Median       3Q      Max
## -3.1656   0.1157   0.1157   0.7409   0.9794
##
## Coefficients:
##                                     Estimate Std. Error z value Pr(>|z|)
## (Intercept)                            5.004      1.003   4.987 6.12e-07 ***
## TreatmentOverlapping                  -3.773      1.028  -3.671 0.000241 ***
## TreatmentNon-overlapping              -3.851      1.025  -3.757 0.000172 ***
## MW_absent                             11.562    799.849   0.014 0.988467
## TreatmentOverlapping:MW_absent       -12.307    799.849  -0.015 0.987724
## TreatmentNon-overlapping:MW_absent   -12.022    799.849  -0.015 0.988008
## ---
## Signif. codes:  0 '***' 0.001 '**' 0.01 '*' 0.05 '.' 0.1 ' ' 1
##
## (Dispersion parameter for binomial family taken to be 1)
##
##     Null deviance: 388.19  on 437  degrees of freedom
## Residual deviance: 323.55  on 432  degrees of freedom
```

```
## AIC: 335.55
##
## Number of Fisher Scoring iterations: 15
```

```r
#checking model fit
check_mod_AT1 <- simulateResiduals(fittedModel = Attempt_mod1,n=1000, plot=T)
```

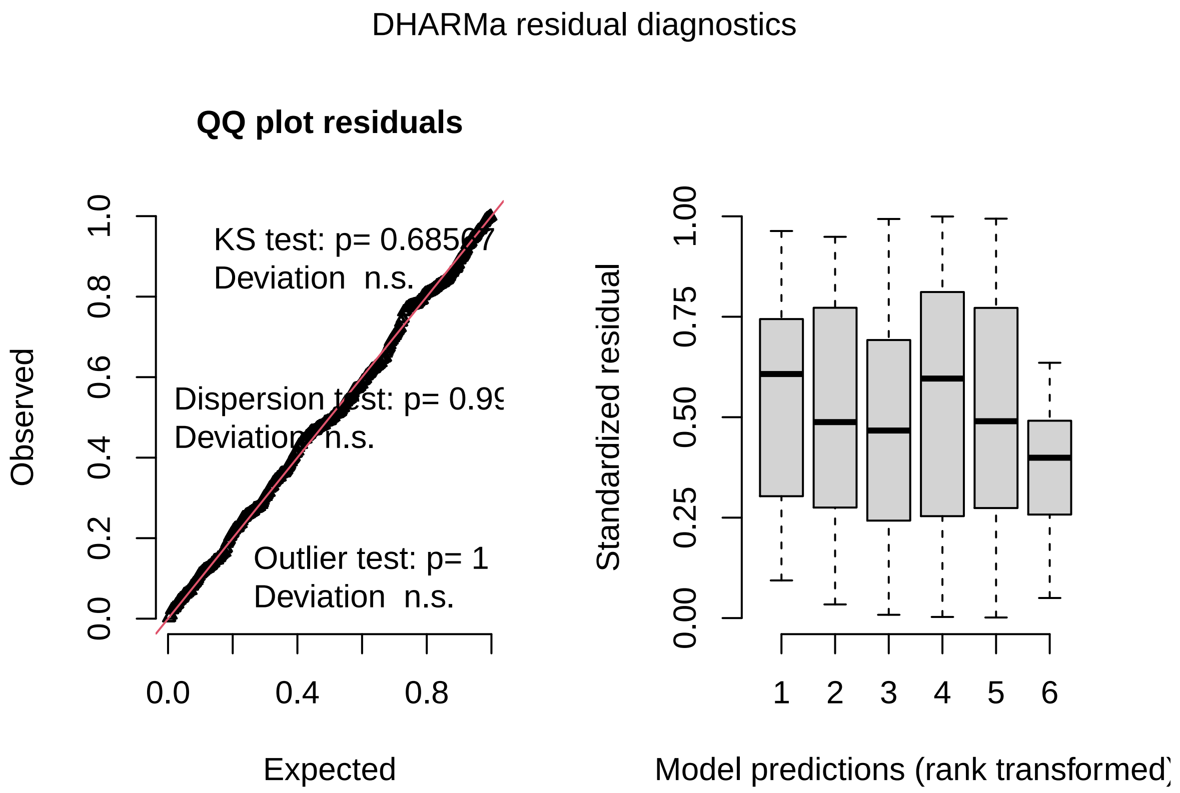


```r
testDispersion(Attempt_mod1)
```

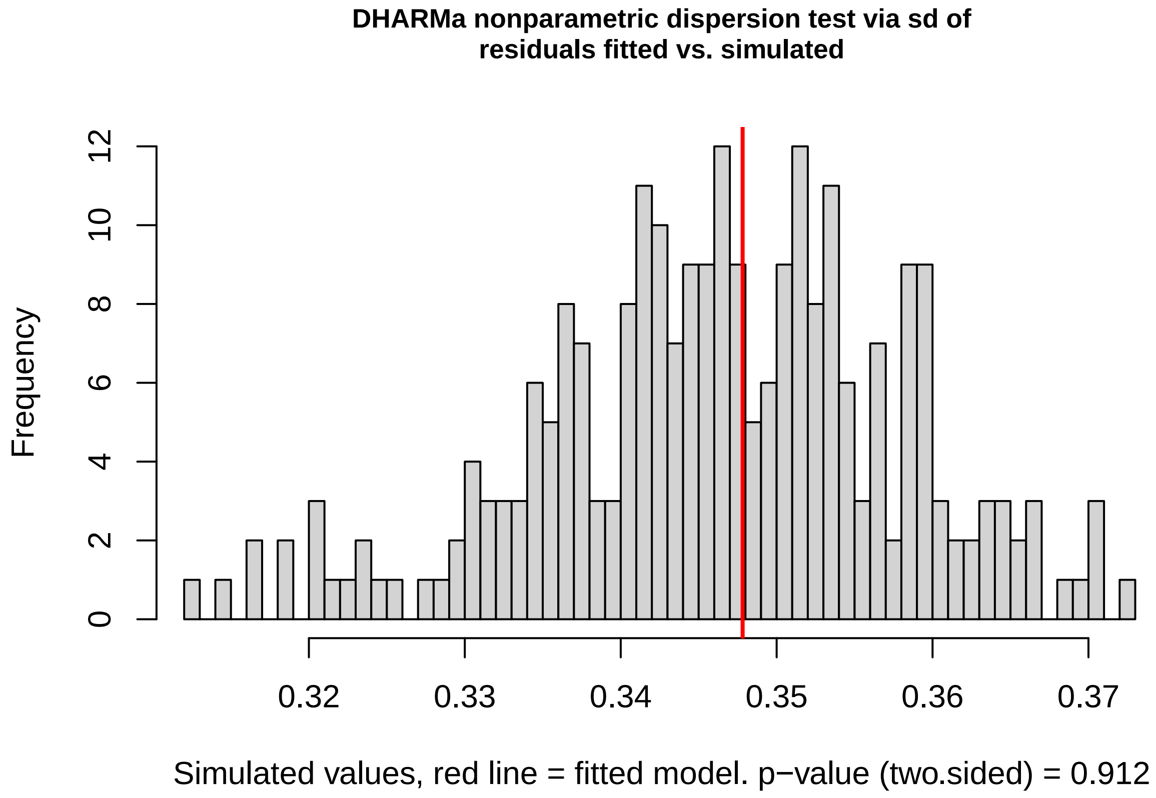


```
##
##  DHARMa nonparametric dispersion test via sd of residuals fitted vs.
##  simulated
##
## data:  simulationOutput
## ratioObsSim = 1.0045, p-value = 0.912
## alternative hypothesis: two.sided
```

```
#Interaction plot for prey reward --> perfect separation in Ambient condition
set_theme(base=theme_classic())
plot_model(Attempt_mod1, type="int", transform="plogis", colors="ipsum")+
labs(caption="Interaction between treatment and the presence of a prey reward on the board. We find
no difference in probability of attempting capture across treatments with or without a prey item.",
title=element_blank())+theme(axis.title.x=element_blank())
```

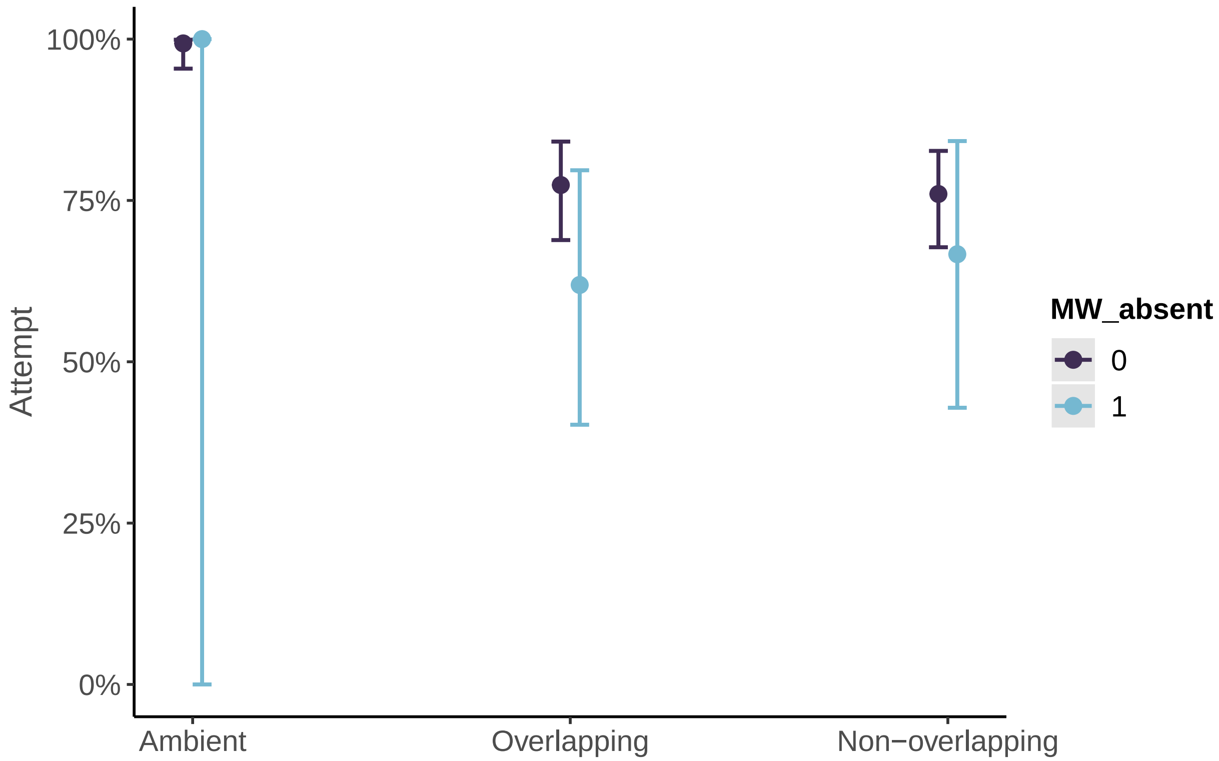

Interaction between treatment and the presence of a prey reward on the board. We find no difference in probability of attempting capture across treatments with or without a prey item.

```
###Model testing the effect of bat ID on trial outcome###
Attempt_mod2<-glm(Attempt~Treatment*Bat, family=binomial(link="logit"), data=dat1)
summary(Attempt_mod2)
```

```
##
## Call:
## glm(formula = Attempt ~ Treatment * Bat, family = binomial(link = "logit"),
##     data = dat1)
##
## Deviance Residuals:
##      Min        1Q    Median        3Q       Max
## -2.81790   0.00013   0.19518   0.70896   0.90052
##
## Coefficients:
##                                 Estimate Std. Error z value Pr(>|z|)
## (Intercept)                    1.857e+01  8.960e+02   0.021    0.983
## TreatmentOverlapping          -1.731e+01  8.960e+02  -0.019    0.985
## TreatmentNon-overlapping      -1.787e+01  8.960e+02  -0.020    0.984
## Bat2                          -1.461e+01  8.960e+02  -0.016    0.987
## Bat3                           1.628e-08  1.267e+03   0.000    1.000
## TreatmentOverlapping:Bat2      1.461e+01  8.960e+02   0.016    0.987
## TreatmentNon-overlapping:Bat2  1.577e+01  8.960e+02   0.018    0.986
## TreatmentOverlapping:Bat3     -4.261e-01  1.267e+03   0.000    1.000
## TreatmentNon-overlapping:Bat3  2.973e-01  1.267e+03   0.000    1.000
##
## (Dispersion parameter for binomial family taken to be 1)
```

```
##
##     Null deviance: 388.19  on 437  degrees of freedom
## Residual deviance: 317.85  on 429  degrees of freedom
## AIC: 335.85
##
## Number of Fisher Scoring iterations: 17
```

```r
#checking model fit
check_mod_AT2 <- simulateResiduals(fittedModel = Attempt_mod2,n=1000, plot=T)
```

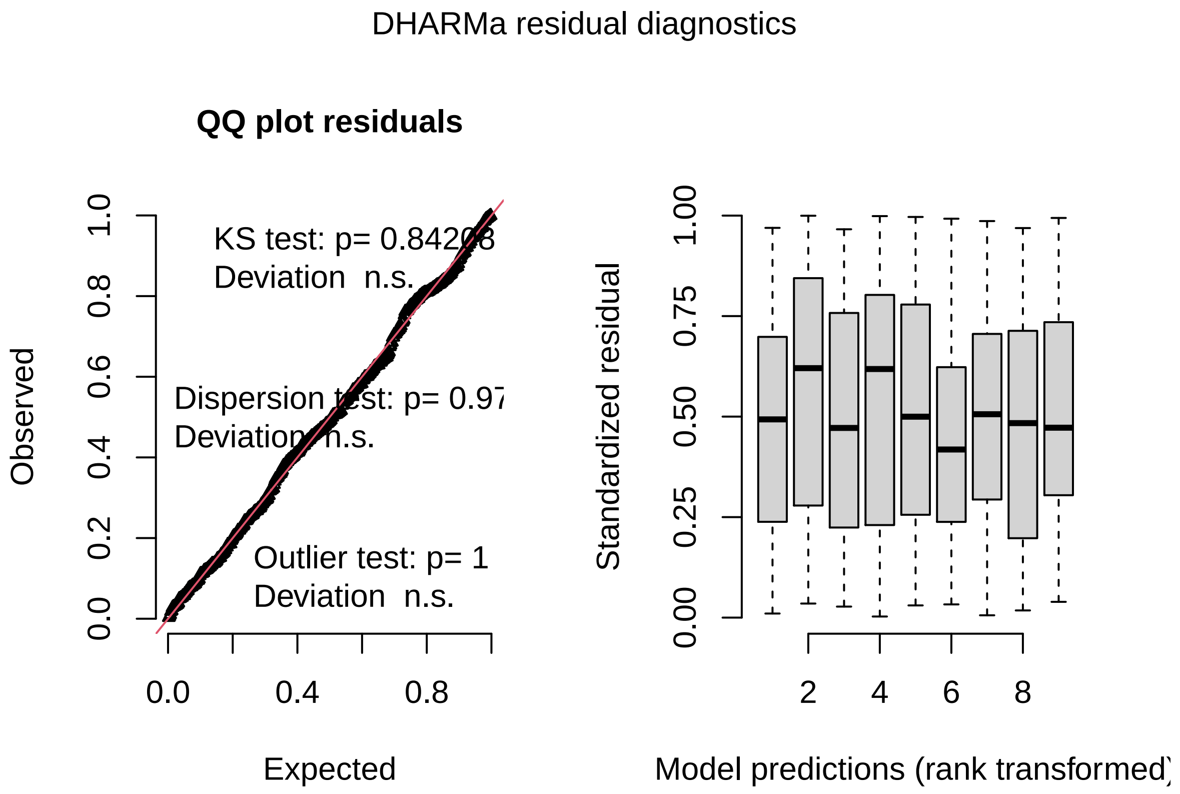


```r
testDispersion(Attempt_mod2)
```

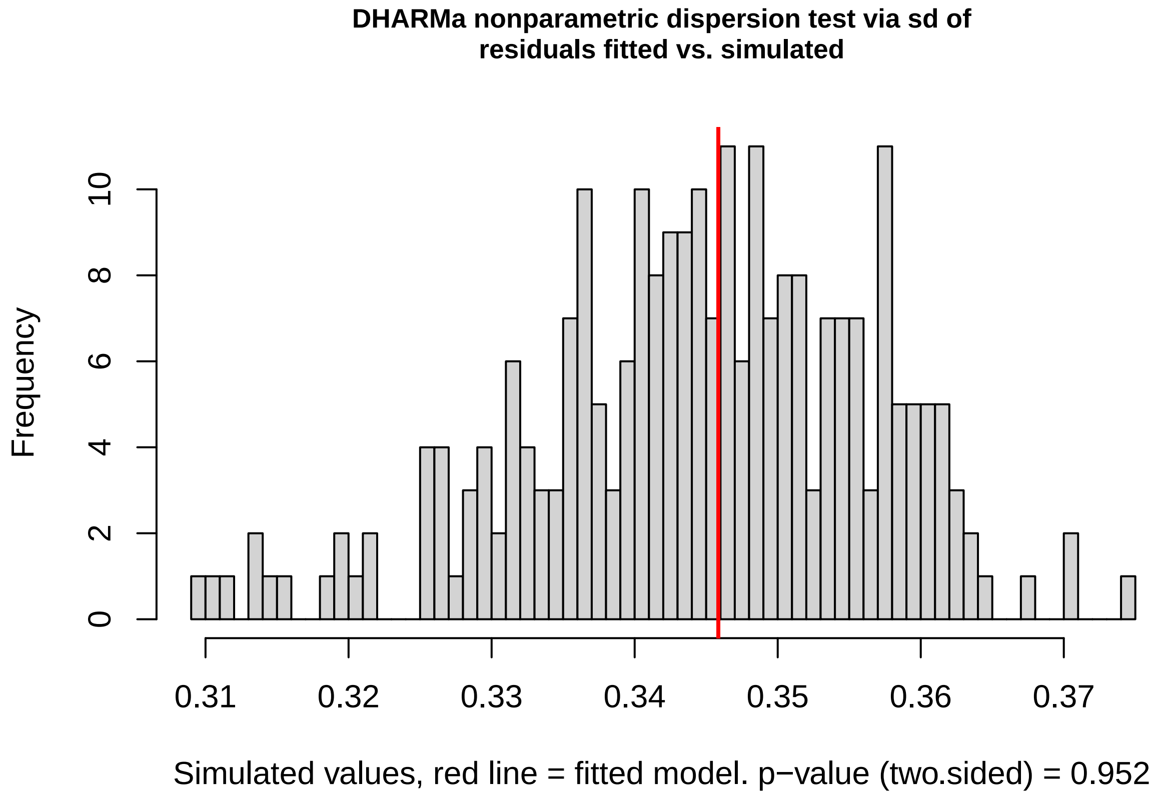


```
##
##  DHARMa nonparametric dispersion test via sd of residuals fitted vs.
##  simulated
##
## data:  simulationOutput
## ratioObsSim = 1.004, p-value = 0.952
## alternative hypothesis: two.sided
```

```r
#Interaction plot for bat ID
plot_model(Attempt_mod2, type="int", transform="plogis", colors="system", ci.lvl=0.95)+
  labs(caption="Interaction between treatment type and bat ID. Bats do not differ from each other acros
       title=element_blank())+theme(axis.title.x=element_blank())
```

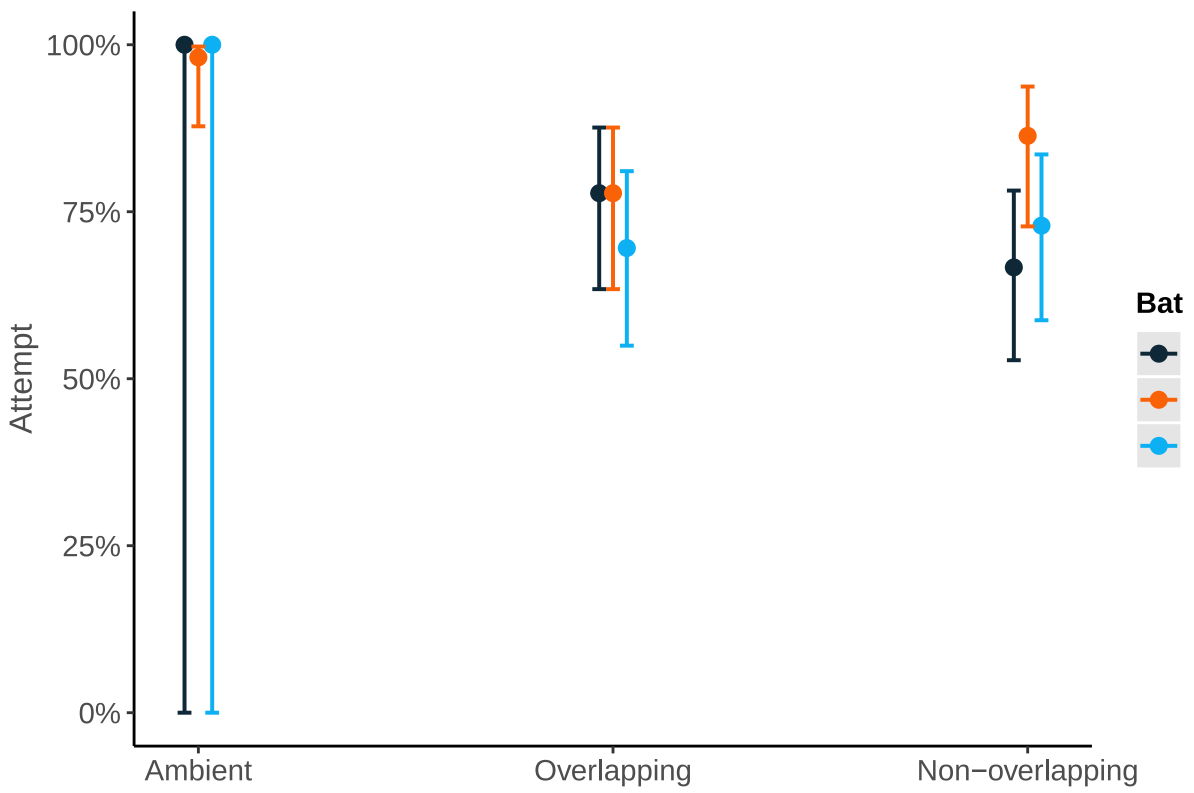

Interaction between treatment type and bat ID. Bats do not differ from each other across treatments.

```
###Model testing the effect of experimental Night on trial outcome###
Attempt_mod3<-glm(Attempt~Treatment*Night, family=binomial(link="logit"), data=dat1)

summary(Attempt_mod3)
```

```
##
## Call:
## glm(formula = Attempt ~ Treatment * Night, family = binomial(link = "logit"),
##     data = dat1)
##
## Deviance Residuals:
##     Min       1Q   Median       3Q      Max
## -2.5308   0.0000   0.2881   0.6117   1.2516
##
## Coefficients:
##                                 Estimate Std. Error z value Pr(>|z|)
## (Intercept)                       154.37   10817.18   0.014    0.989
## TreatmentOverlapping             -155.33   10817.18  -0.014    0.989
## TreatmentNon-overlapping         -154.87   10817.18  -0.014    0.989
## Night                             -15.18    1081.72  -0.014    0.989
## TreatmentOverlapping:Night         15.59    1081.72   0.014    0.988
## TreatmentNon-overlapping:Night     15.51    1081.72   0.014    0.989
##
## (Dispersion parameter for binomial family taken to be 1)
##
##     Null deviance: 388.19  on 437  degrees of freedom
```

```
## Residual deviance: 276.08  on 432  degrees of freedom
## AIC: 288.08
##
## Number of Fisher Scoring iterations: 21
```

```r
#checking model fit
check_mod_AT3 <- simulateResiduals(fittedModel = Attempt_mod3,n=1000, plot=T)
```

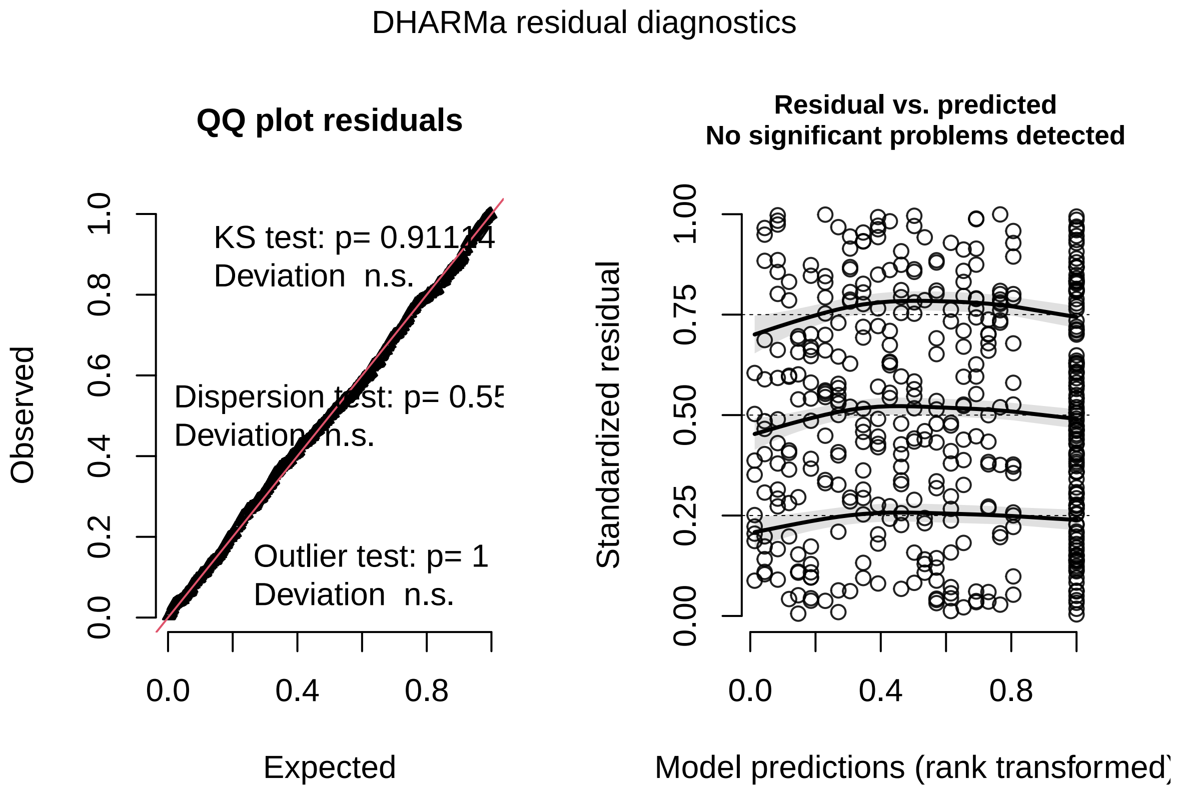


```r
testDispersion(Attempt_mod3)
```

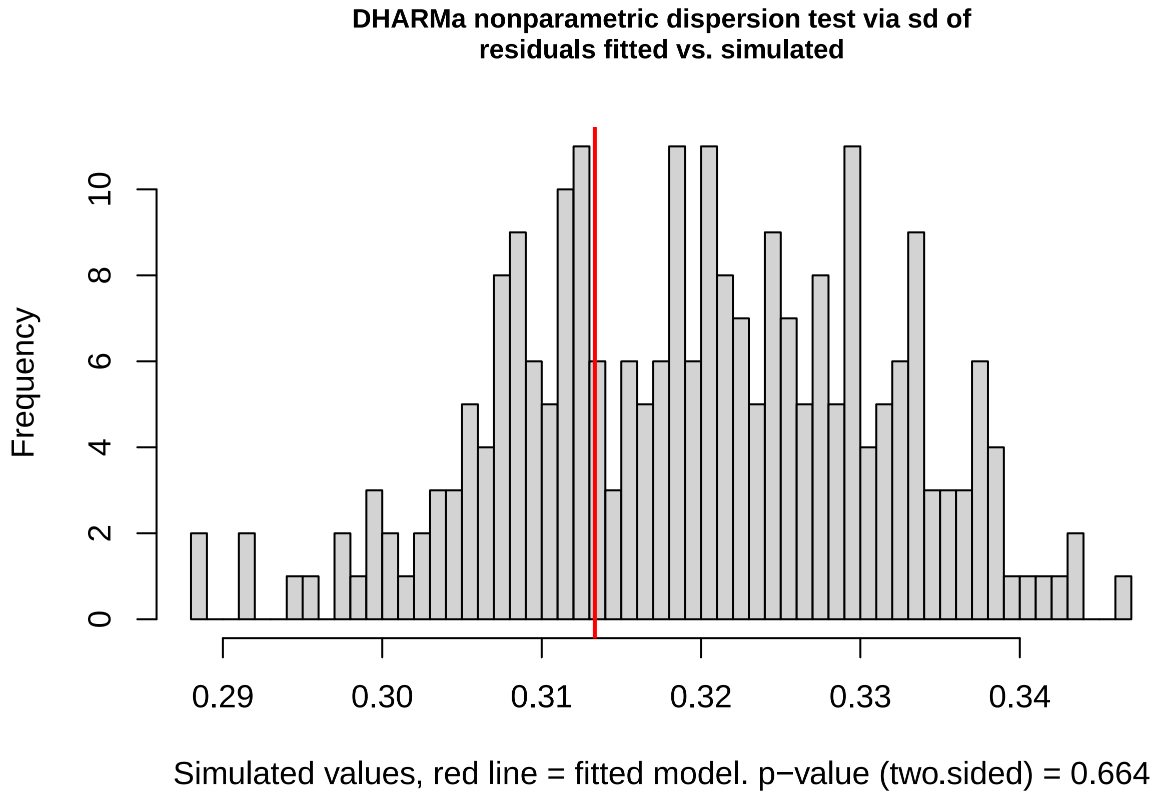


```
##
##  DHARMa nonparametric dispersion test via sd of residuals fitted vs.
##  simulated
##
## data:  simulationOutput
## ratioObsSim = 0.98019, p-value = 0.664
## alternative hypothesis: two.sided
```

##Success Models

The following models test the effect of noise on bats' ability to successfully localize prey. That is, the probability of landing on the correct speaker playing the prey cue

```
#subsetting to keep only trials where bats made an attempt
dat2 <-droplevels(subset(dat1, Attempt!="0"))

###Model testing the effect of prey reward on trial outcome###
success_mod1<-glmer(Success~Treatment*MW_absent+(1|Bat), family=binomial(link="logit"), data=dat2)
summary(success_mod1)
```

```
## Generalized linear mixed model fit by maximum likelihood (Laplace
##   Approximation) [glmerMod]
##  Family: binomial  ( logit )
## Formula: Success ~ Treatment * MW_absent + (1 | Bat)
##    Data: dat2
##
##      AIC      BIC   logLik deviance df.resid
##    348.1    375.4   -167.0    334.1      360
```

```
##
## Scaled residuals:
##     Min      1Q  Median      3Q     Max
## -4.1653 -0.6547  0.2112  0.2557  1.6422
##
## Random effects:
##  Groups Name        Variance Std.Dev.
##  Bat    (Intercept) 0.05228  0.2287
## Number of obs: 367, groups:  Bat, 3
##
## Fixed effects:
##                                    Estimate Std. Error z value Pr(>|z|)
## (Intercept)                          2.8962     0.3891   7.444  9.8e-14 ***
## TreatmentOverlapping                -3.5742     0.4304  -8.304  < 2e-16 ***
## TreatmentNon-overlapping            -3.7192     0.4297  -8.656  < 2e-16 ***
## MW_absent                           -1.6997     0.8889  -1.912  0.05585 .
## TreatmentOverlapping:MW_absent       3.2017     1.1004   2.909  0.00362 **
## TreatmentNon-overlapping:MW_absent   2.7888     1.0864   2.567  0.01026 *
## ---
## Signif. codes:  0 '***' 0.001 '**' 0.01 '*' 0.05 '.' 0.1 ' ' 1
##
## Correlation of Fixed Effects:
##             (Intr) TrtmnO TrtmN- MW_bsn TO:MW_
## TrtmntOvrlp -0.801
## TrtmntNn-vr -0.802  0.727
## MW_absent   -0.393  0.356  0.356
## TrtmntO:MW_  0.319 -0.397 -0.290 -0.810
## TrtmnN-:MW_  0.316 -0.286 -0.394 -0.811  0.655
```

```
#checking model fit
check_mod_Suc1 <- simulateResiduals(fittedModel = success_mod1,n=1000, plot=T)
```

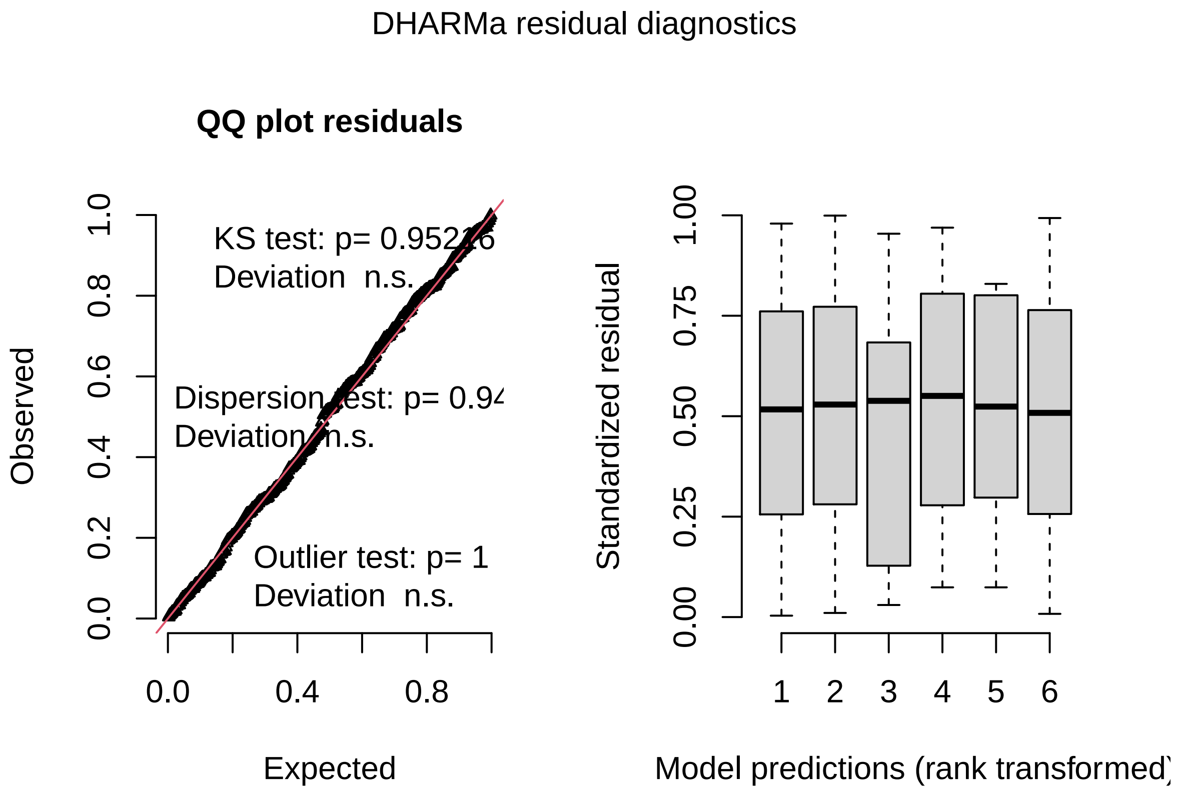


```
testDispersion(success_mod1)
```

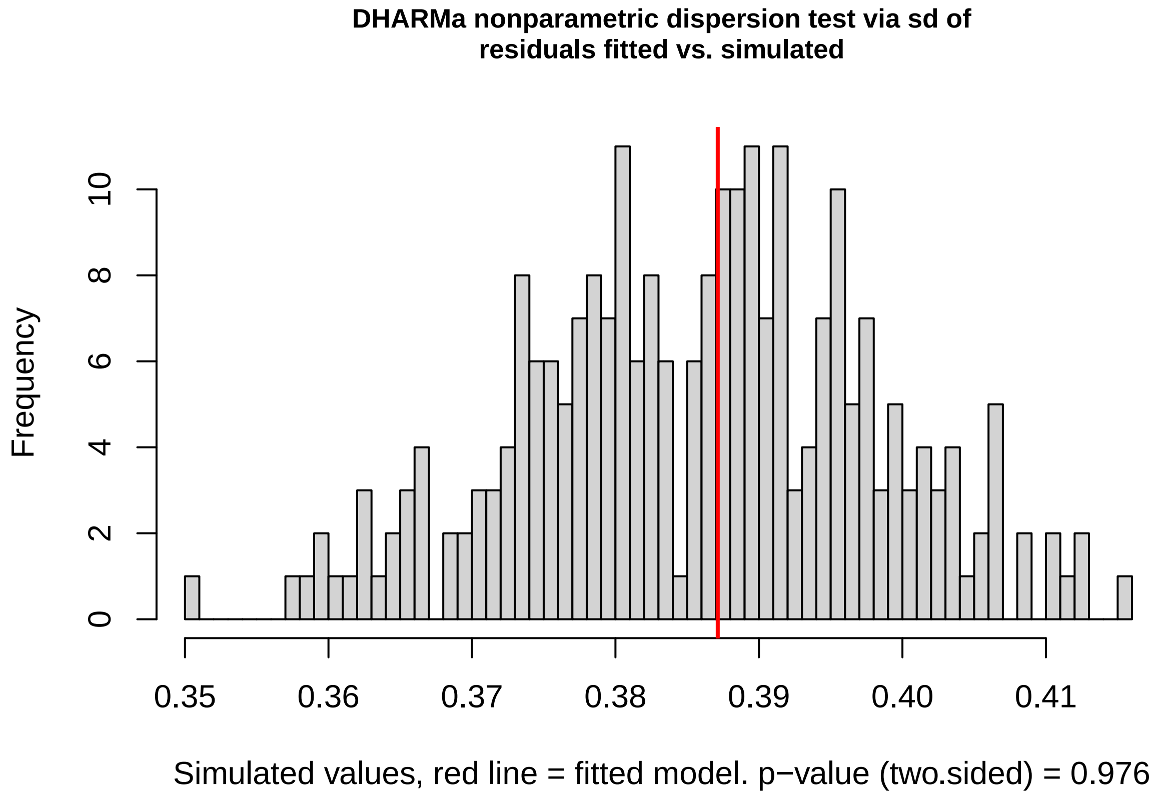


```
##
##  DHARMa nonparametric dispersion test via sd of residuals fitted vs.
##  simulated
##
## data:  simulationOutput
## ratioObsSim = 1.0036, p-value = 0.976
## alternative hypothesis: two.sided
```

```
#Interaction plot for prey reward on Success
plot_model(success_mod1, type="int", transform="plogis", colors="ipsum")+
labs(caption="Interaction between treatment type and presence of prey reward on the board.
                                                                  There is no difference i
```

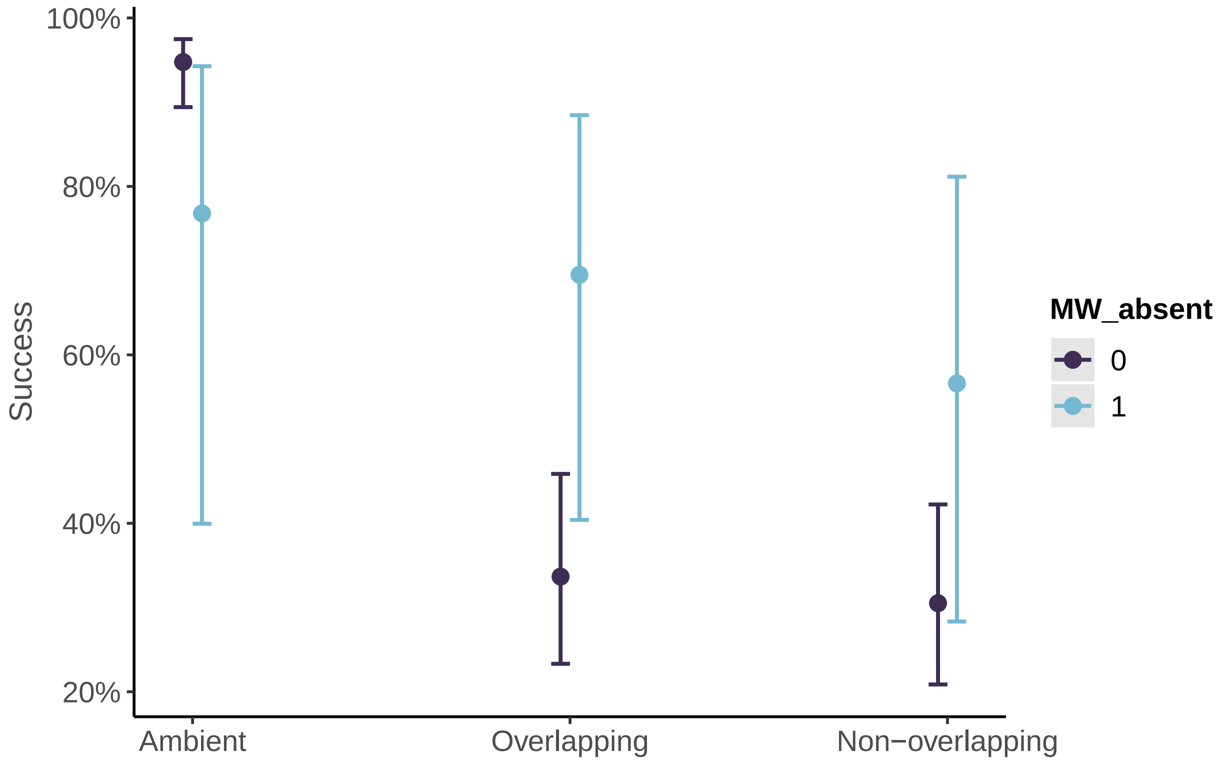

Interaction between treatment type and presence of prey reward on the board.
There is no difference in probability of success with prey on or off the board

```r
###Model testing the effect of Night and bat ID on success###
success_mod2<-glmer(Success~Night+(1|Bat), data=dat2, family=binomial(link="logit"))
summary(success_mod2)
```

```
## Generalized linear mixed model fit by maximum likelihood (Laplace
##   Approximation) [glmerMod]
##  Family: binomial  ( logit )
## Formula: Success ~ Night + (1 | Bat)
##    Data: dat2
##
##      AIC      BIC   logLik deviance df.resid
##    497.3    509.0   -245.7    491.3      364
##
## Scaled residuals:
##     Min      1Q  Median      3Q     Max
## -1.3356 -1.2118  0.7640  0.8169  0.8505
##
## Random effects:
##  Groups Name        Variance Std.Dev.
##  Bat    (Intercept) 0.01581  0.1258
## Number of obs: 367, groups:  Bat, 3
##
## Fixed effects:
##             Estimate Std. Error z value Pr(>|z|)
## (Intercept)  0.38066    0.27061   1.407    0.160
## Night        0.01009    0.04042   0.250    0.803
```

```
##
## Correlation of Fixed Effects:
##       (Intr)
## Night -0.878
```

```r
#checking model fit
check_mod_Suc2 <- simulateResiduals(fittedModel = success_mod2,n=1000, plot=T)
```

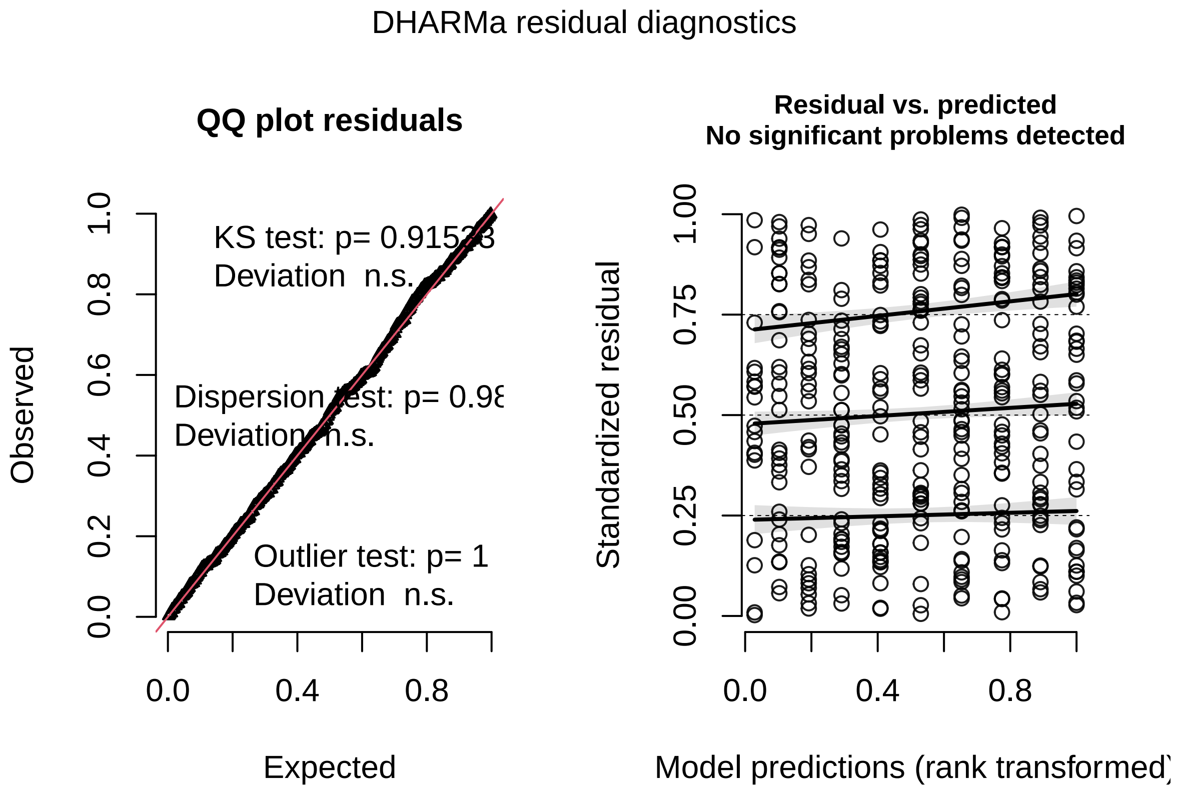


```r
hist(check_mod_Suc2)
```

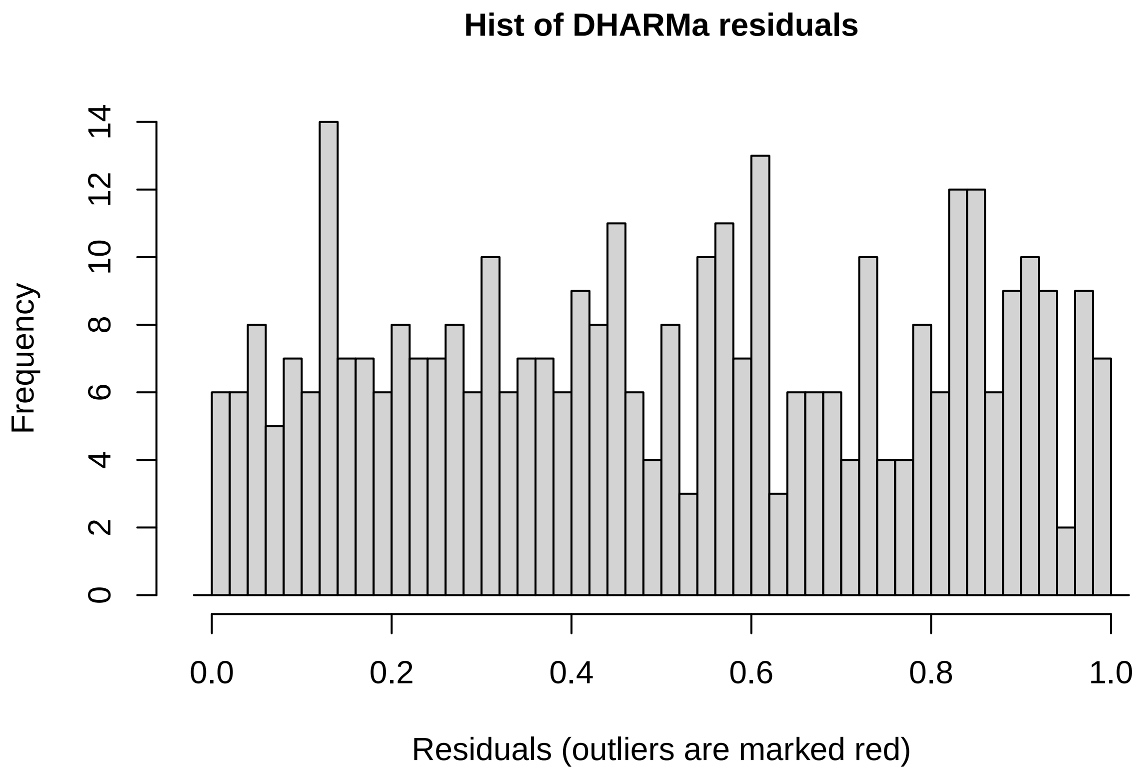


```r
testDispersion(success_mod2)
```

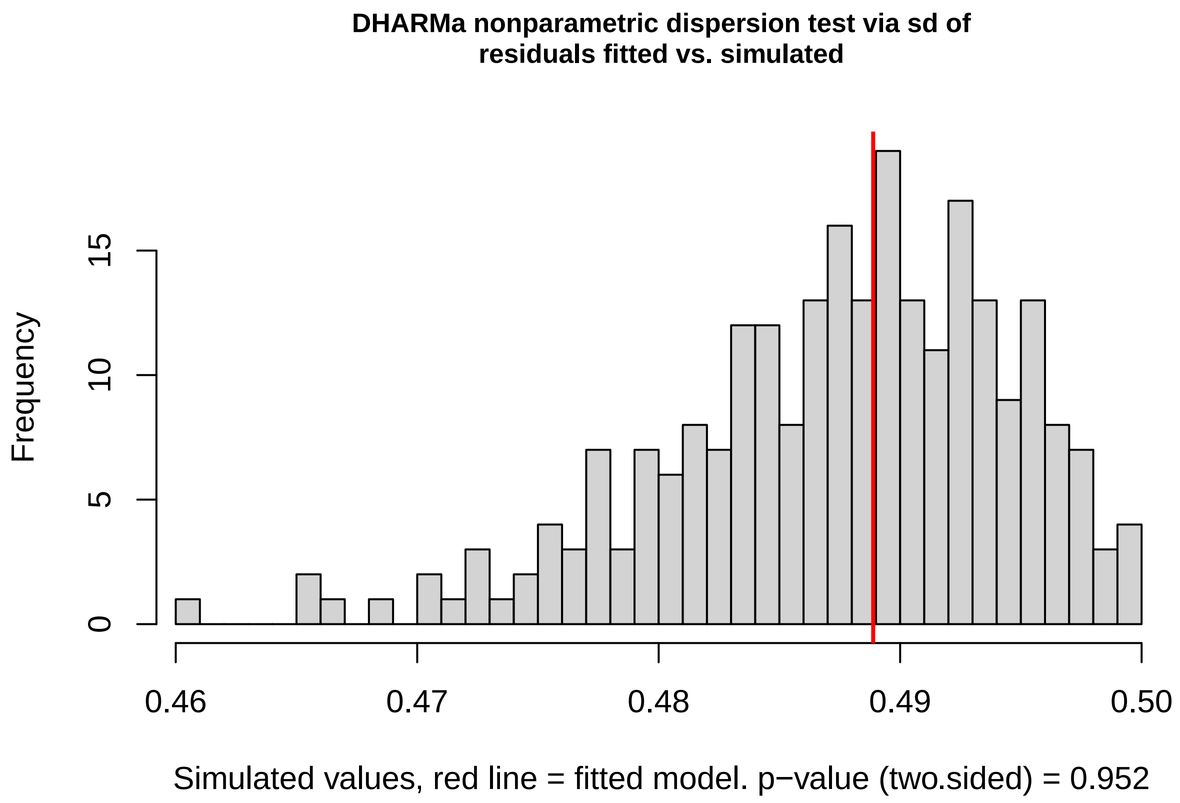


```
##
##  DHARMa nonparametric dispersion test via sd of residuals fitted vs.
##  simulated
##
## data:  simulationOutput
## ratioObsSim = 1.0031, p-value = 0.952
## alternative hypothesis: two.sided
```

```
#Plot of the effect of Night and bat ID on success
plot_model(success_mod2, type="pred", terms=c("Night", "Bat"), pred.type="re", transform="plogis",
           colors="system",dot.size=1.2,line.size = 0.8, ci.lvl=0.95, show.data=TRUE, jitter=.05)+
  scale_y_continuous(limits=c(-.05,1.05),breaks=c(0,.2,.4,.6,.8,1))+
  scale_x_continuous(breaks=c(1, 4, 7, 10),
```

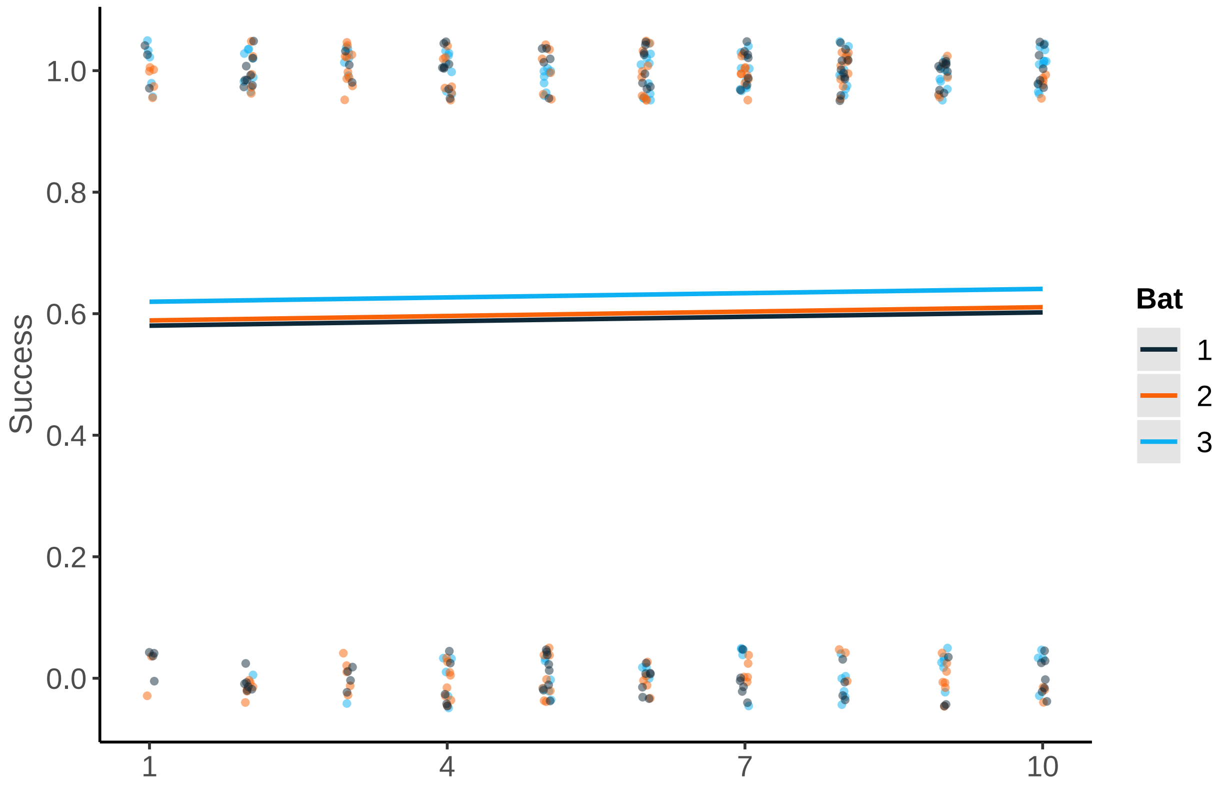

Probability of success does not change across experimental night for all bats

```r
###Model testing the effect of Treatment on Sucess###
success_mod3<-glmer(Success~Treatment+(1|Bat), data=dat2, family=binomial(link="logit"))

summary(success_mod3)
```

```
## Generalized linear mixed model fit by maximum likelihood (Laplace
##   Approximation) [glmerMod]
##  Family: binomial  ( logit )
## Formula: Success ~ Treatment + (1 | Bat)
##    Data: dat2
##
##      AIC      BIC   logLik deviance df.resid
##    354.0    369.6   -173.0    346.0      363
##
## Scaled residuals:
##     Min      1Q  Median      3Q     Max
## -3.7972 -0.7203  0.2291  0.2806  1.5412
##
## Random effects:
##  Groups Name        Variance Std.Dev.
##  Bat    (Intercept) 0.05595  0.2365
## Number of obs: 367, groups:  Bat, 3
##
## Fixed effects:
##                            Estimate Std. Error z value Pr(>|z|)
## (Intercept)                  2.7185     0.3562   7.631 2.32e-14 ***
```

```
## TreatmentOverlapping      -3.1976     0.3879  -8.243  < 2e-16 ***
## TreatmentNon-overlapping  -3.4067     0.3894  -8.749  < 2e-16 ***
## ---
## Signif. codes:  0 '***' 0.001 '**' 0.01 '*' 0.05 '.' 0.1 ' ' 1
##
## Correlation of Fixed Effects:
##             (Intr) TrtmnO
## TrtmntOvrlp -0.784
## TrtmntNn-vr -0.783  0.720
```

```
#Getting confidence intervals
load("easyPredCI.Rdata")
pframe <- data.frame(Treatment=factor(levels(dat2$Treatment),
                                levels=levels(dat2$Treatment)))
cpred1 <- predict(success_mod3,re.form=NA,newdata=pframe,type="response")
cpred1.CI <- easyPredCI(success_mod3,pframe)


#checking model fit
check_mod_Suc3 <- simulateResiduals(fittedModel = success_mod3,n=1000, plot=T)
```

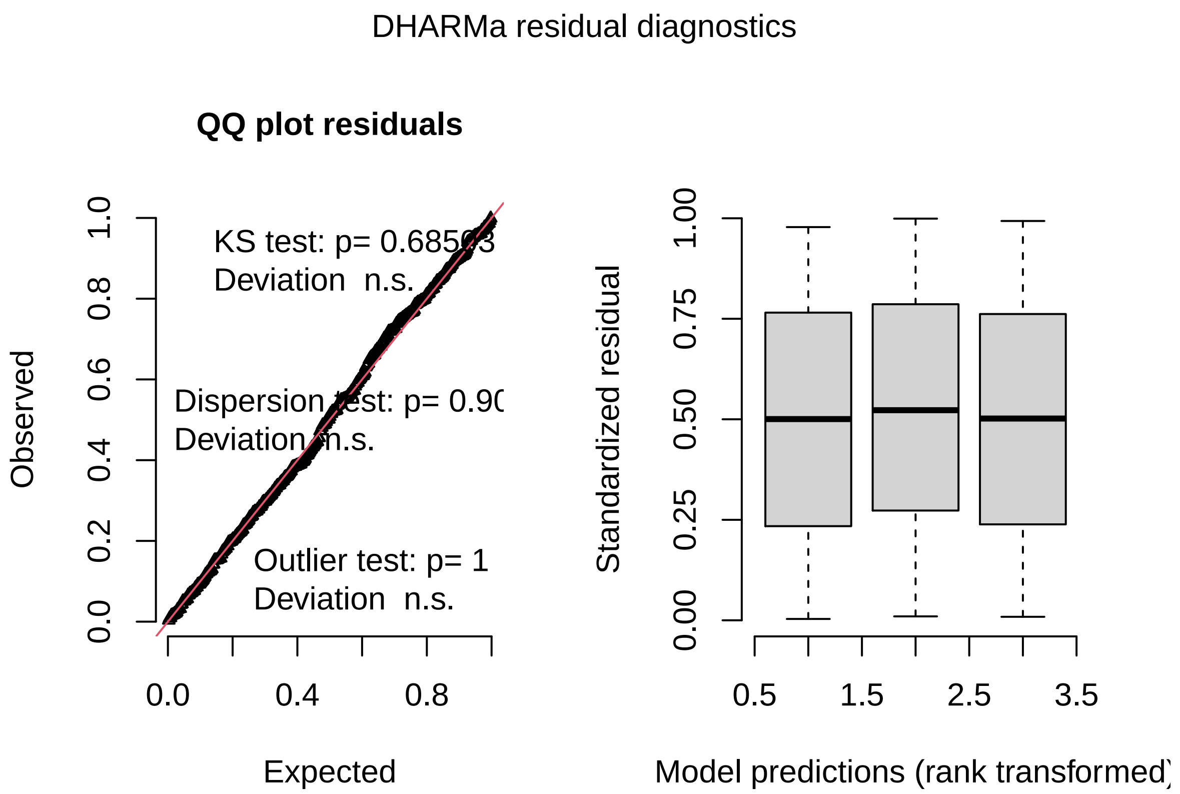


```
testDispersion(success_mod3)
```

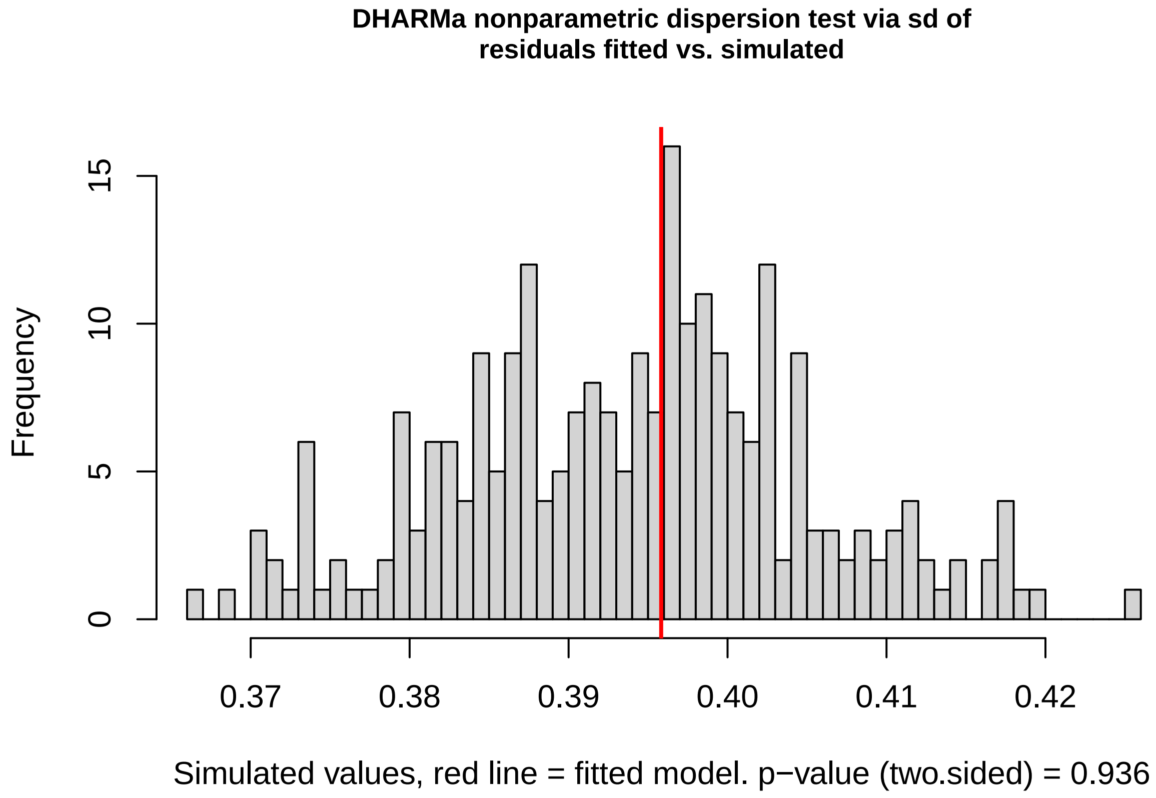


```
##
##  DHARMa nonparametric dispersion test via sd of residuals fitted vs.
##  simulated
##
## data:  simulationOutput
## ratioObsSim = 1.005, p-value = 0.936
## alternative hypothesis: two.sided
```

##Search time models

The following models test the effect of noise on bats' latency to make a foraging decision. That is, the time from prey signal start that it takes for the bat to land on a selected speaker on the board.

```
#cleaning the data set for search time models - i.e., getting rid of trial without start time
dat2<-droplevels(subset(dat2, Search_time!="NA"))

###Model testing the effect of prey reward on trial outcome###
ST_mod1<-glmer(Search_time~Treatment*MW_absent+(1|Bat), family=Gamma(link="log"), data=dat2)

summary(ST_mod1)
```

```
## Generalized linear mixed model fit by maximum likelihood (Laplace
##   Approximation) [glmerMod]
##  Family: Gamma  ( log )
## Formula: Search_time ~ Treatment * MW_absent + (1 | Bat)
##    Data: dat2
##
##      AIC      BIC   logLik deviance df.resid
```

```
##    2219.0   2250.2  -1101.5   2203.0      358
##
## Scaled residuals:
##     Min      1Q  Median      3Q     Max
## -1.2217 -0.5599 -0.2576  0.1758  7.6221
##
## Random effects:
##  Groups   Name        Variance Std.Dev.
##  Bat      (Intercept) 0.02276  0.1509
##  Residual             0.62875  0.7929
## Number of obs: 366, groups:  Bat, 3
##
## Fixed effects:
##                                    Estimate Std. Error t value Pr(>|z|)
## (Intercept)                         1.38837    0.11710  11.856  < 2e-16 ***
## TreatmentOverlapping                1.41304    0.08898  15.880  < 2e-16 ***
## TreatmentNon-overlapping            1.26069    0.08702  14.488  < 2e-16 ***
## MW_absent                           0.27178    0.22671   1.199  0.23060
## TreatmentOverlapping:MW_absent     -0.79038    0.29976  -2.637  0.00837 **
## TreatmentNon-overlapping:MW_absent  0.14559    0.30437   0.478  0.63241
## ---
## Signif. codes:  0 '***' 0.001 '**' 0.01 '*' 0.05 '.' 0.1 ' ' 1
##
## Correlation of Fixed Effects:
##             (Intr) TrtmnO TrtmN- MW_bsn TO:MW_
## TrtmntOvrlp -0.285
## TrtmntNn-vr -0.292  0.384
## MW_absent   -0.109  0.143  0.145
## TrtmntO:MW_  0.083 -0.295 -0.111 -0.756
## TrtmnN-:MW_  0.079 -0.104 -0.280 -0.744  0.561
```

```
#model checking
check_mod_ST1 <- simulateResiduals(fittedModel = ST_mod1,n=1000, plot=T)
```

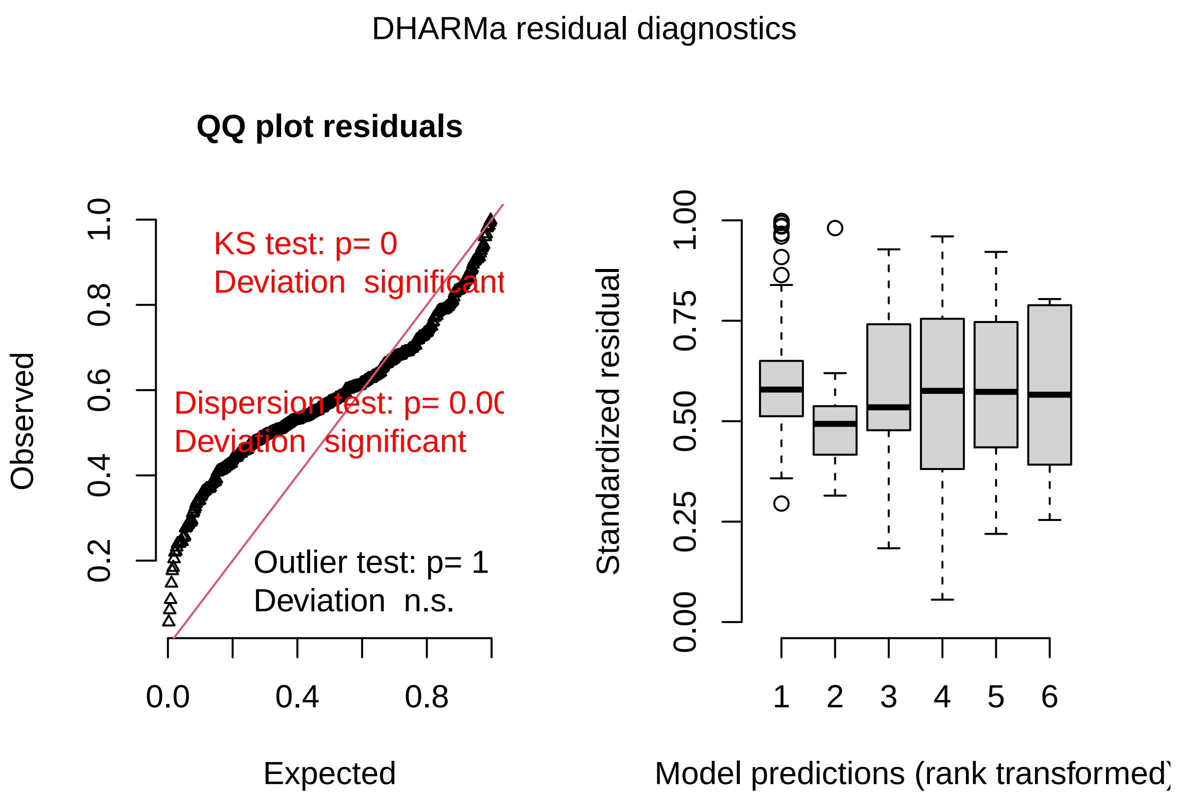


```
testDispersion(ST_mod1)
```

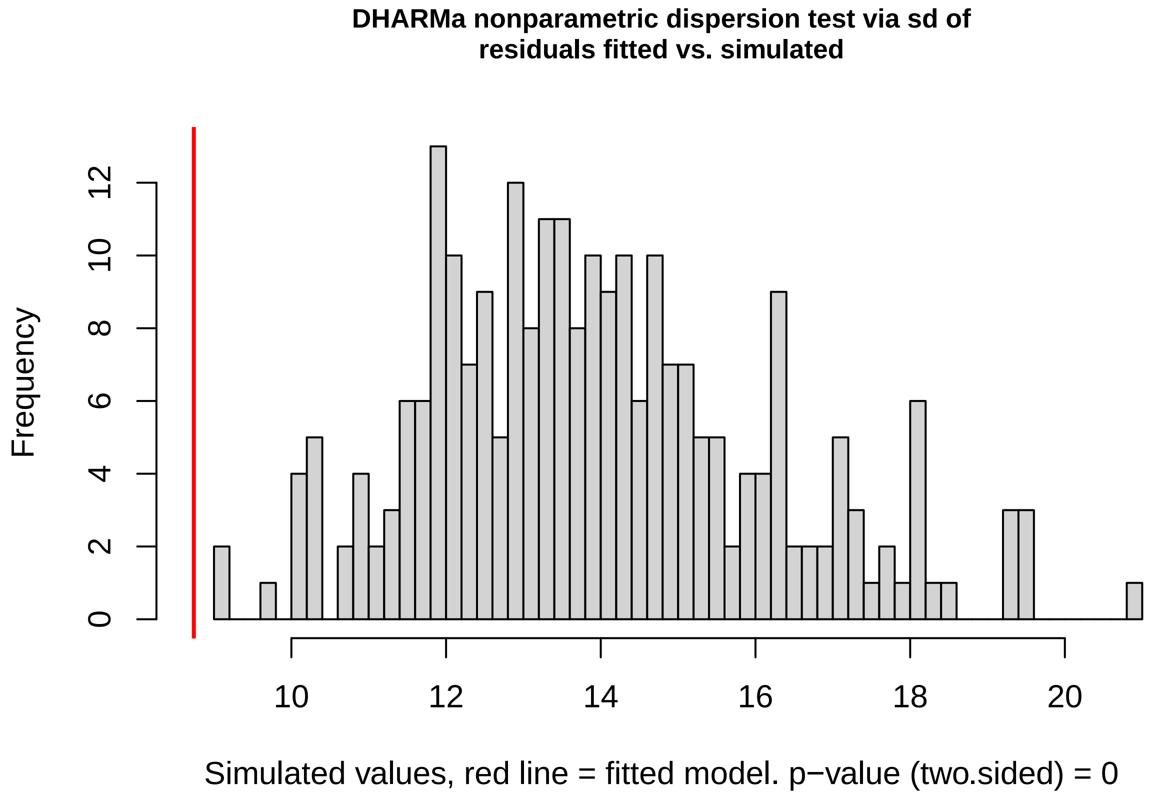


```
##
##  DHARMa nonparametric dispersion test via sd of residuals fitted vs.
##  simulated
##
## data:  simulationOutput
## ratioObsSim = 0.62702, p-value < 2.2e-16
## alternative hypothesis: two.sided
```

```
#Interaction plot for prey reward on Search Time
plot_model(ST_mod1, type="int", transform="exp", colors="ipsum")+
labs(caption="Interaction of treatment and presence of prey reward on the board.
                                                                      There is no difference in search t
  ylab("Search time (ms)")
```

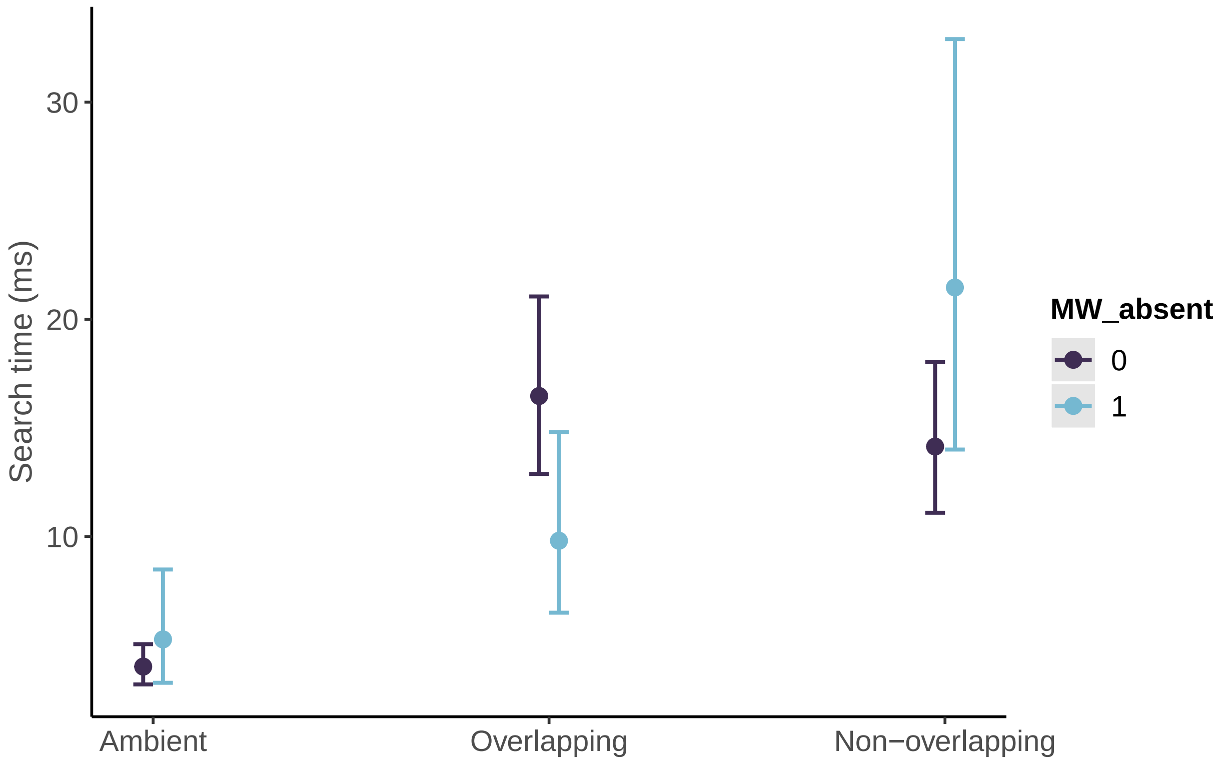

Interaction of treatment and presence of prey reward on the board.
There is no difference in search time within treatments with prey on or off the board.

```r
#Testing effect of Night
ST_mod2<-glmer(Search_time~Night+(1|Bat), data=dat2, family=Gamma(link="log"))
summary(ST_mod2)
```

```
## Generalized linear mixed model fit by maximum likelihood (Laplace
##   Approximation) [glmerMod]
##  Family: Gamma  ( log )
## Formula: Search_time ~ Night + (1 | Bat)
##    Data: dat2
##
##      AIC      BIC   logLik deviance df.resid
##   2453.9   2469.5  -1222.9   2445.9      362
##
## Scaled residuals:
##     Min      1Q  Median      3Q     Max
## -0.9606 -0.6906 -0.4660  0.3293  4.1725
##
## Random effects:
##  Groups   Name        Variance Std.Dev.
##  Bat      (Intercept) 0.01251  0.1119
##  Residual             0.99737  0.9987
## Number of obs: 366, groups:  Bat, 3
##
## Fixed effects:
##             Estimate Std. Error t value Pr(>|z|)
## (Intercept)  2.26879    0.13273  17.093   <2e-16 ***
```

```
## Night        0.01328    0.01815    0.732    0.464
## ---
## Signif. codes:  0 '***' 0.001 '**' 0.01 '*' 0.05 '.' 0.1 ' ' 1
##
## Correlation of Fixed Effects:
##       (Intr)
## Night -0.807
```

```
#checking model fit
check_mod_ST2 <- simulateResiduals(fittedModel = ST_mod2,n=1000, plot=T)
```

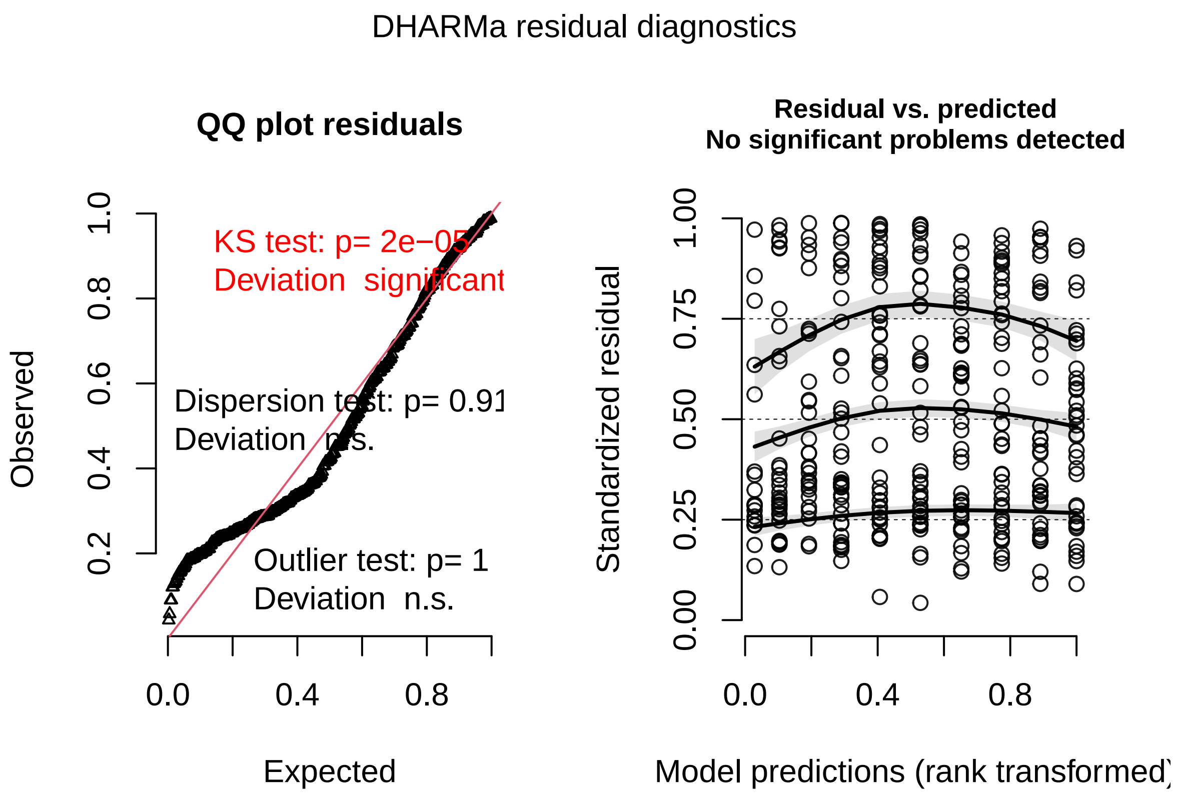


```
testDispersion(ST_mod2)
```

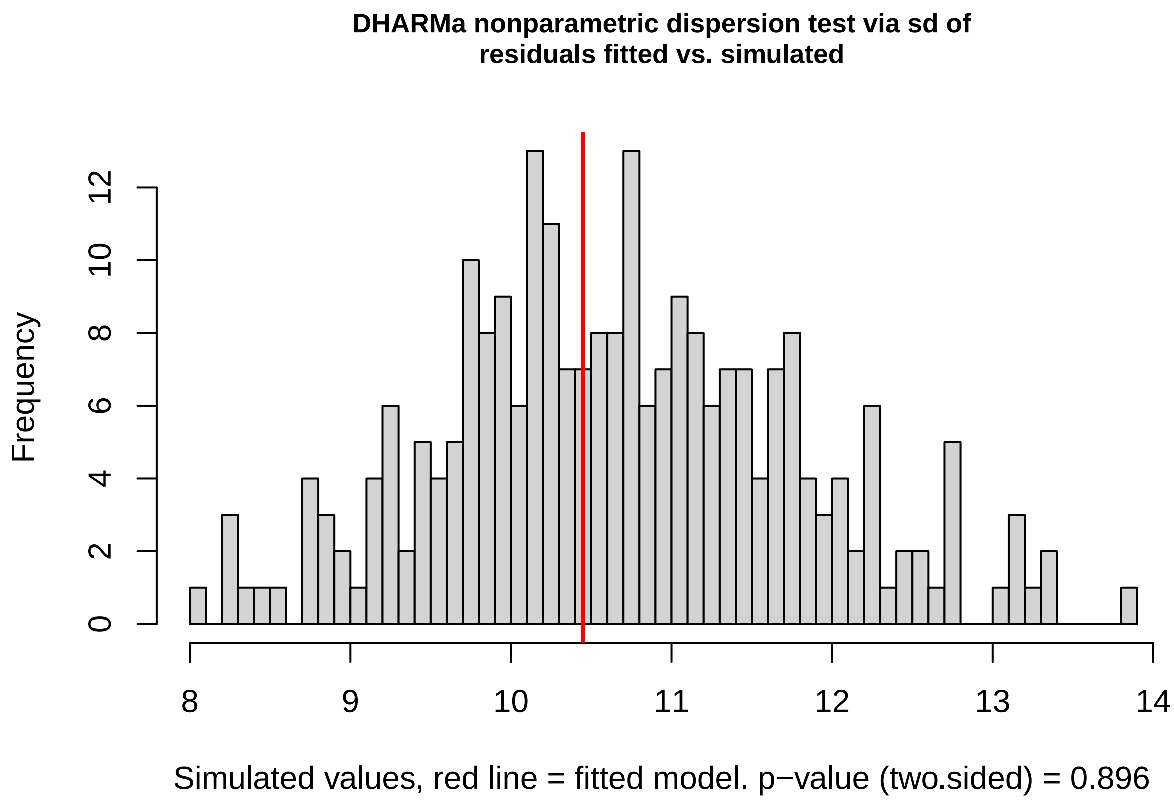


```
##
##  DHARMa nonparametric dispersion test via sd of residuals fitted vs.
##  simulated
##
## data:  simulationOutput
## ratioObsSim = 0.97804, p-value = 0.896
## alternative hypothesis: two.sided
```

```
#Plot of effect of Night and bat ID on Search time
plot_model(ST_mod2, type="pred", terms=c("Night", "Bat"), pred.type="re", transform="exp",
           colors="system",dot.size=1.2,line.size = .8, ci.lvl=0.95, show.data=TRUE)+labs(y="Search Tim
                                                                                             title=element_
```

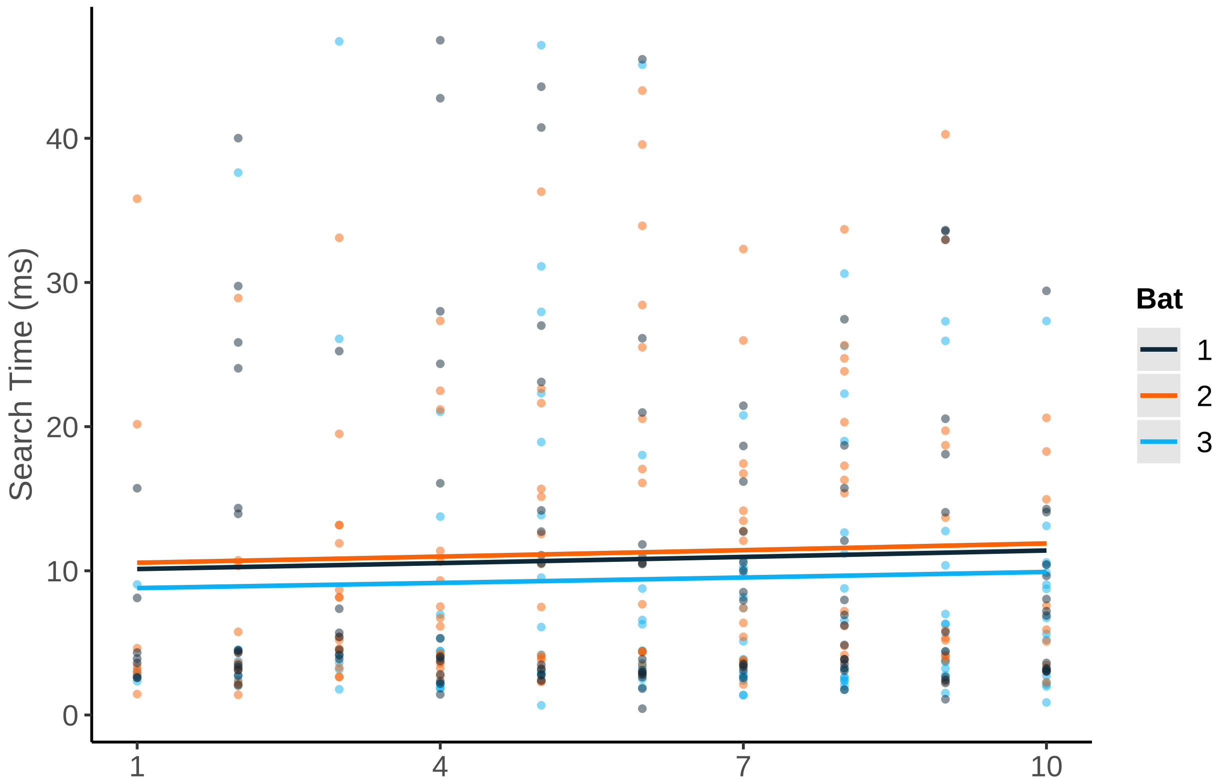

Search time does not change across experimental night for all bats

```
###Model testing the effect of Treatment on Search Time###

ST_mod3<-glmer(Search_time~Treatment+(1|Bat), data=dat2, family=Gamma(link="log"))
summary(ST_mod3)
```

```
## Generalized linear mixed model fit by maximum likelihood (Laplace
##   Approximation) [glmerMod]
##  Family: Gamma  ( log )
## Formula: Search_time ~ Treatment + (1 | Bat)
##    Data: dat2
##
##      AIC      BIC   logLik deviance df.resid
##   2225.2   2244.7  -1107.6   2215.2      361
##
## Scaled residuals:
##     Min      1Q  Median      3Q     Max
## -1.2022 -0.5512 -0.2775  0.1436  7.4298
##
## Random effects:
##  Groups   Name        Variance Std.Dev.
##  Bat      (Intercept) 0.02073  0.1440
##  Residual             0.65151  0.8072
## Number of obs: 366, groups:  Bat, 3
##
## Fixed effects:
##                           Estimate Std. Error t value Pr(>|z|)
```

```
## (Intercept)               1.40714    0.11100   12.68   <2e-16 ***
## TreatmentOverlapping      1.34116    0.08561   15.67   <2e-16 ***
## TreatmentNon-overlapping  1.29780    0.08445   15.37   <2e-16 ***
## ---
## Signif. codes:  0 '***' 0.001 '**' 0.01 '*' 0.05 '.' 0.1 ' ' 1
##
## Correlation of Fixed Effects:
##             (Intr) TrtmnO
## TrtmntOvrlp -0.304
## TrtmntNn-vr -0.311  0.402
```

```r
#checking model fit
check_ST_mod3 <- simulateResiduals(fittedModel = ST_mod3, n=1000, plot=T)
```

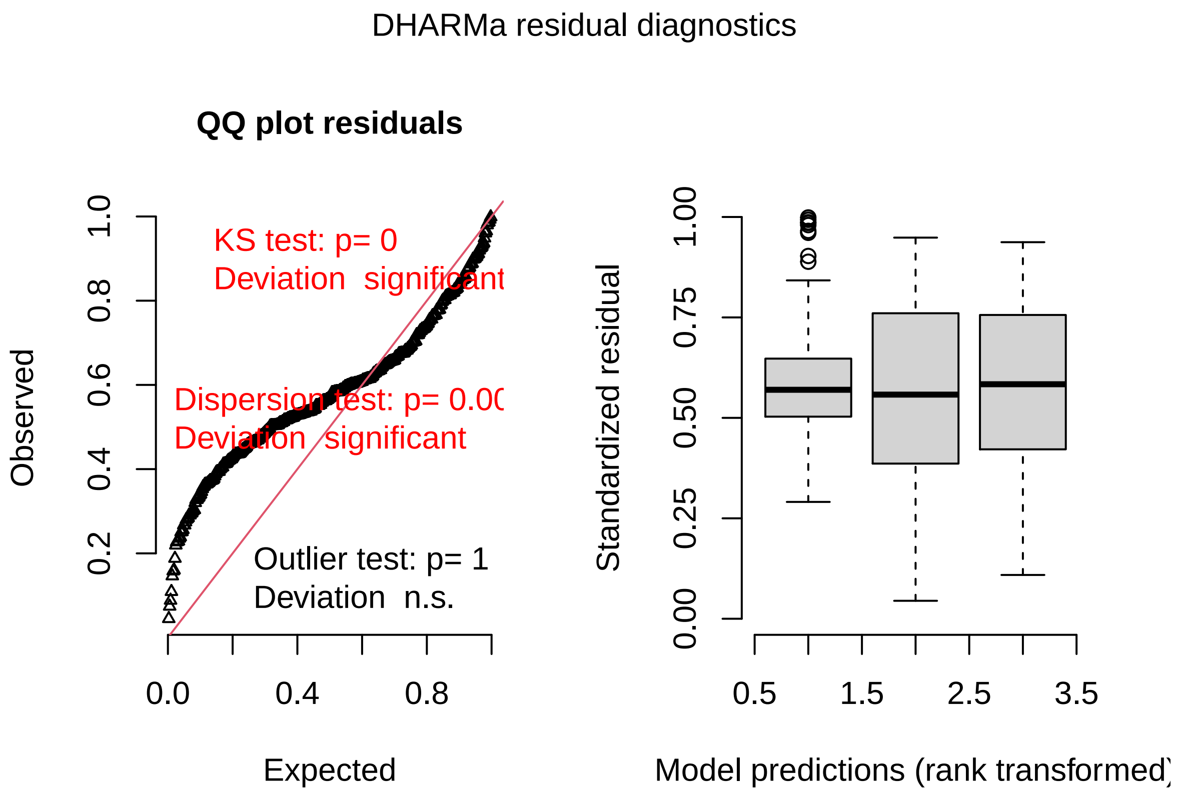


```r
testDispersion(ST_mod3)
```

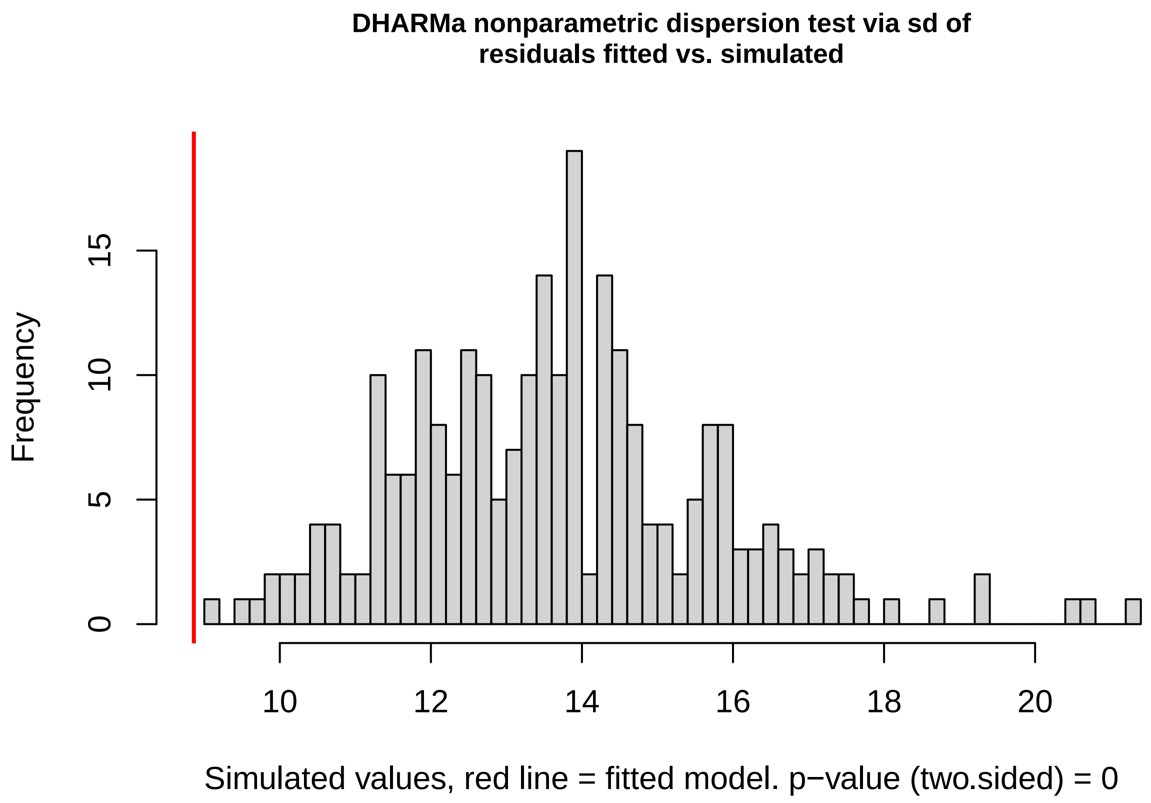


```
##
##  DHARMa nonparametric dispersion test via sd of residuals fitted vs.
##  simulated
##
## data:  simulationOutput
## ratioObsSim = 0.64686, p-value < 2.2e-16
## alternative hypothesis: two.sided
```

```
#getting confidence intervals
emmeans(ST_mod3, "Treatment", transform="log", type="response")
```

```
##  Treatment       response    SE  df asymp.LCL asymp.UCL
##  Ambient             4.08 0.453 Inf      3.29      5.08
##  Overlapping        15.62 1.839 Inf     12.40     19.67
##  Non-overlapping    14.95 1.746 Inf     11.90     18.80
##
## Confidence level used: 0.95
## Intervals are back-transformed from the log scale
```

##Echolocation models

The following models test the effect of noise on bats' echolocation parameters, including average interpulse interval (IPI) and dominant frequency.

```
#reording Treatment levels
dat3$Treatment<-factor(dat3$Treatment, levels=c("Ambient", "Overlapping", "Non-overlapping"))

#making data numerical
```

```r
dat3$IPI<-as.character(dat3$IPI)
dat3$IPI<-as.numeric(dat3$IPI)
dat3$freq_maxdB<-as.character(dat3$freq_maxdB)
dat3$freq_maxdB<-as.numeric(dat3$freq_maxdB)
dat3$MW_absent<-as.factor(dat3$MW_absent)

dat3 <- transform(dat3, UniqueID=match(Video, unique(Video)))

#building an empty matrix
acous <- matrix(ncol=22, nrow=0)

#runnign a loop to calculate the average IPI and dom freq for each trial
for (i in unique(dat3$UniqueID)){

  newdata <- dat3[which(dat3$UniqueID==i & dat3$Attempt=="1"),]
  avg_IPI<-mean(newdata$IPI, na.rm=T)
  avg_dfreq<-mean(newdata$freq_maxdB, na.rm=T)

  x=newdata[1,]

  y=cbind(x, avg_IPI, avg_dfreq)

  acous=rbind(acous,y)

}


#removing NA's
acous<-acous[!is.na(acous$avg_IPI),]
acous<-acous[!is.na(acous$avg_dfreq),]

#converting to ms
acous$avg_IPI<-acous$avg_IPI*1000

###Model testing the effect of prey reward on trial outcome###
IPI_mod1<-lmer(avg_IPI~Treatment*MW_absent+(1|Bat), data=acous)
summary(IPI_mod1)
```

```
## Linear mixed model fit by REML ['lmerMod']
## Formula: avg_IPI ~ Treatment * MW_absent + (1 | Bat)
##    Data: acous
##
## REML criterion at convergence: 1270.4
##
## Scaled residuals:
##     Min      1Q  Median      3Q     Max
## -1.8183 -0.6449 -0.1462  0.4195  2.7271
##
## Random effects:
##  Groups   Name        Variance Std.Dev.
##  Bat      (Intercept)  79.11    8.894
##  Residual             576.56   24.012
## Number of obs: 142, groups:  Bat, 3
##
```

```
## Fixed effects:
##                                   Estimate Std. Error t value
## (Intercept)                         95.918      6.171  15.544
## TreatmentOverlapping               -21.626      5.553  -3.894
## TreatmentNon-overlapping           -20.446      5.127  -3.988
## MW_absent1                         -33.647     17.315  -1.943
## TreatmentOverlapping:MW_absent1     44.525     20.040   2.222
## TreatmentNon-overlapping:MW_absent1  28.957     19.274   1.502
##
## Correlation of Fixed Effects:
##             (Intr) TrtmnO TrtmN- MW_bs1 TO:MW_
## TrtmntOvrlp -0.343
## TrtmntNn-vr -0.372  0.418
## MW_absent1  -0.099  0.111  0.119
## TrtmnO:MW_1  0.083 -0.260 -0.099 -0.865
## TrtmN-:MW_1  0.093 -0.105 -0.258 -0.897  0.775
```

```r
#checking model fit
check_IPI_mod1 <- simulateResiduals(fittedModel = IPI_mod1, n=1000, plot=T)
```

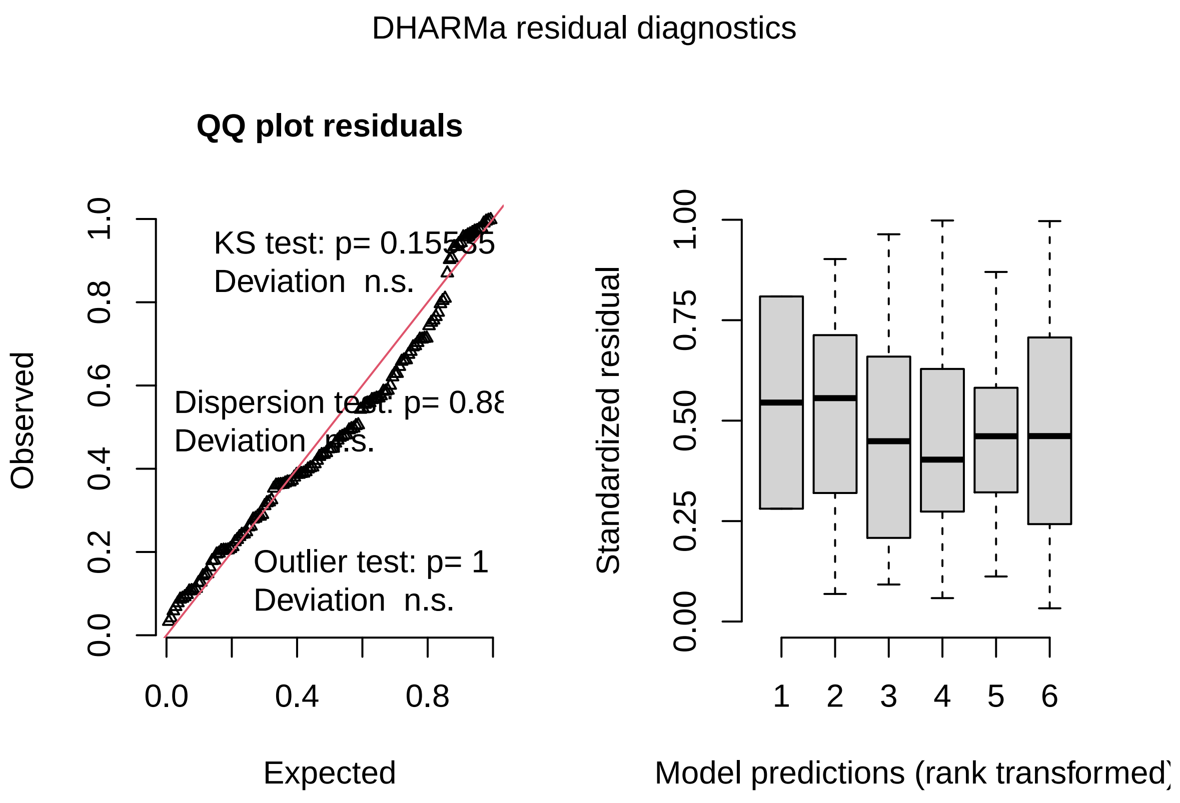


```r
testDispersion(IPI_mod1)
```

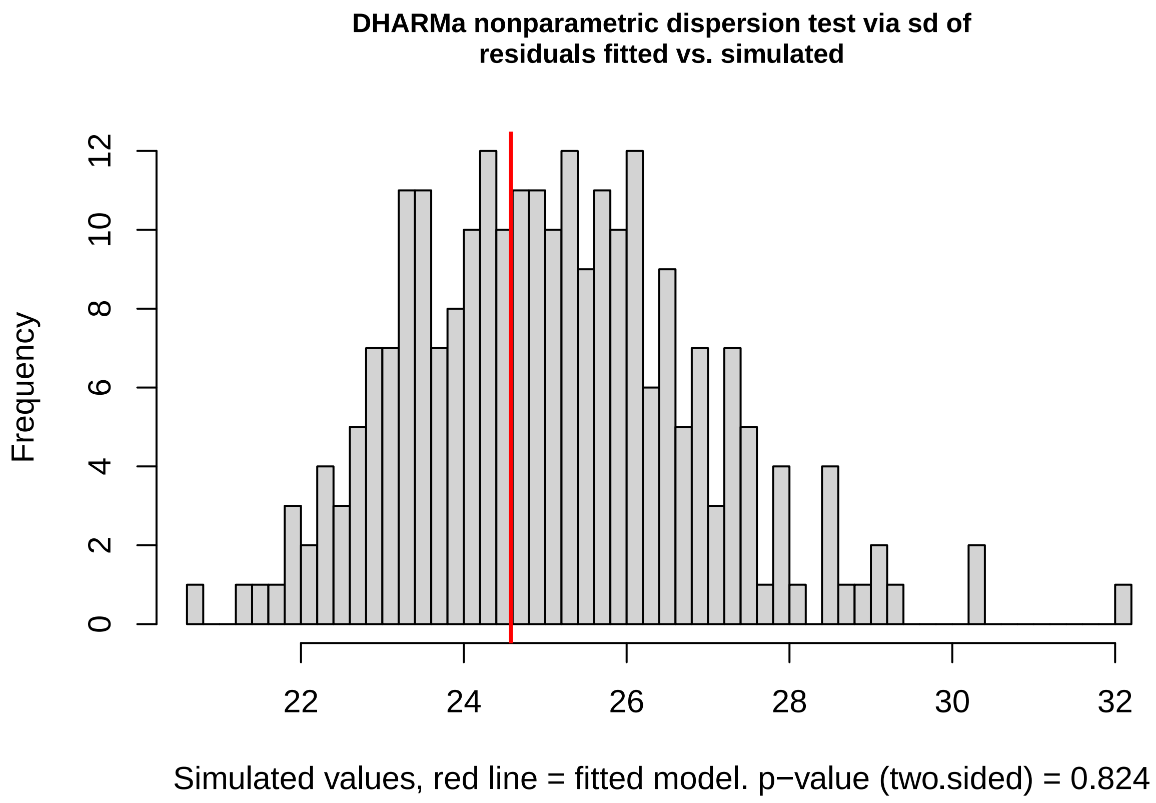


```
##
##  DHARMa nonparametric dispersion test via sd of residuals fitted vs.
##  simulated
##
## data:  simulationOutput
## ratioObsSim = 0.98034, p-value = 0.824
## alternative hypothesis: two.sided
```

```
#Interaction plot for prey reward on avg IPI
plot_model(IPI_mod1, type="int", transform="exp", colors="ipsum")+
labs(y="Avg IPI (ms)",caption="Interaction of treatment and presence of prey reward on the board.
                                                                                          There is r
```

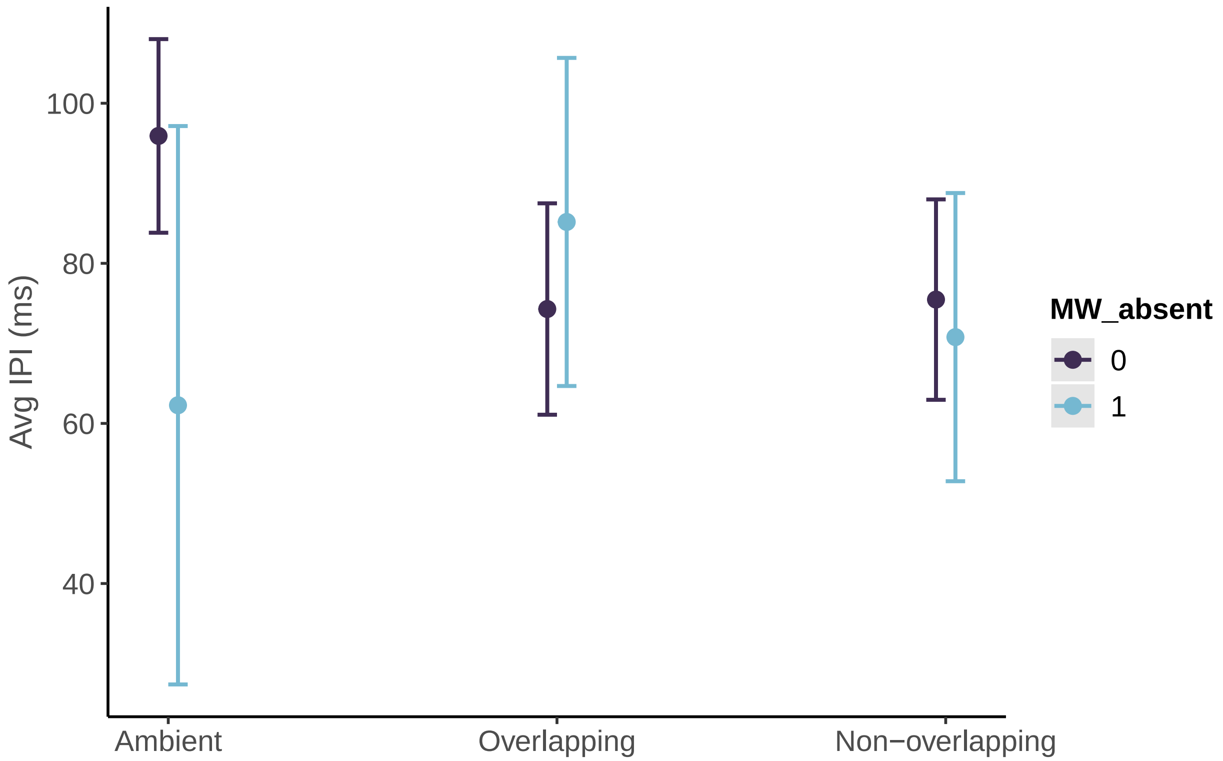

Interaction of treatment and presence of prey reward on the board.
There is no difference of IPI within treatments with prey on or off the board.

```r
###IPI by Night###

IPI_mod2<-glmer.nb(avg_IPI~Night+(1|Bat), data=acous)
summary(IPI_mod2)
```

```
## Generalized linear mixed model fit by maximum likelihood (Laplace
##   Approximation) [glmerMod]
##  Family: Negative Binomial(12.7612)  ( log )
## Formula: avg_IPI ~ Night + (1 | Bat)
##    Data: acous
##
##      AIC      BIC   logLik deviance df.resid
##   1321.4   1333.2   -656.7   1313.4      138
##
## Scaled residuals:
##     Min      1Q  Median      3Q     Max
## -1.7111 -0.7858 -0.1679  0.5111  2.6300
##
## Random effects:
##  Groups Name        Variance Std.Dev.
##  Bat    (Intercept) 0.01192  0.1092
## Number of obs: 142, groups:  Bat, 3
##
## Fixed effects:
##             Estimate Std. Error z value Pr(>|z|)
## (Intercept)  4.421999   0.080899  54.661   <2e-16 ***
```

```
## Night        -0.002702   0.007591  -0.356    0.722
## ---
## Signif. codes:  0 '***' 0.001 '**' 0.01 '*' 0.05 '.' 0.1 ' ' 1
##
## Correlation of Fixed Effects:
##       (Intr)
## Night -0.540
```

```r
#checking model fit
check_IPI_mod2 <- simulateResiduals(fittedModel = IPI_mod2, n=1000, plot=T)
```

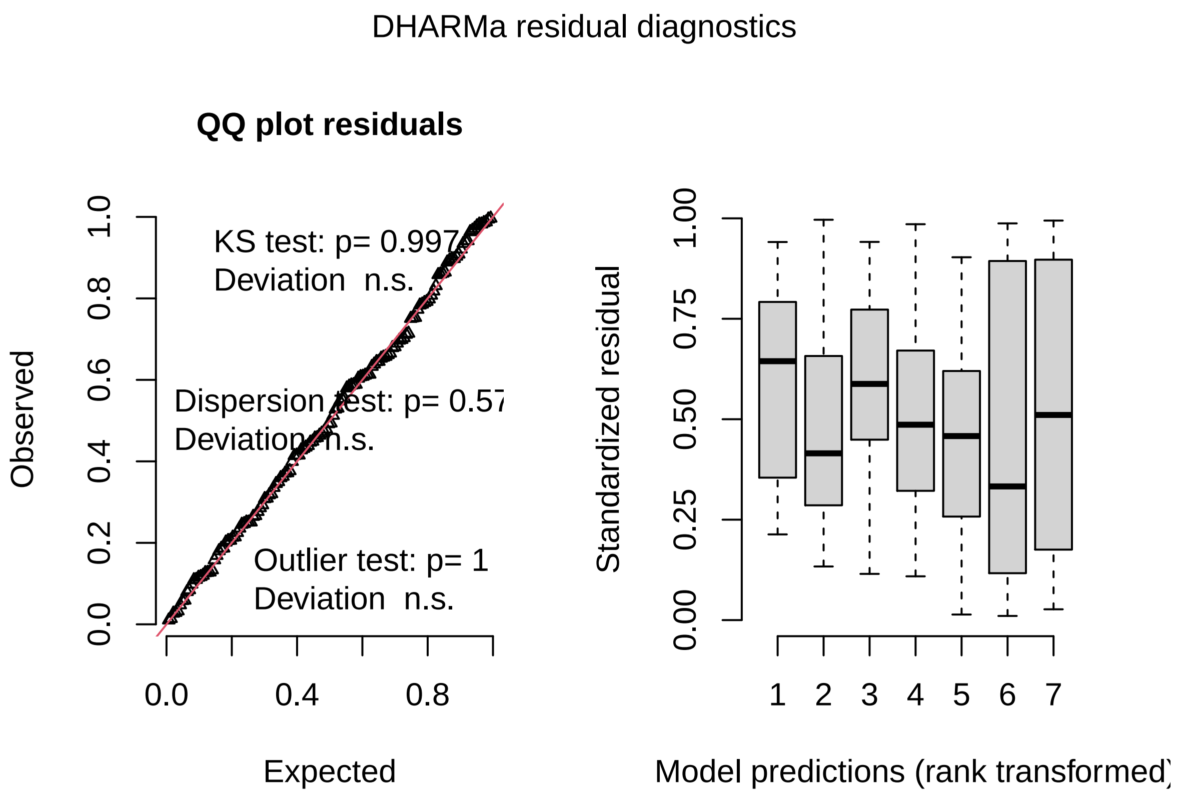


```r
testDispersion(IPI_mod2)
```

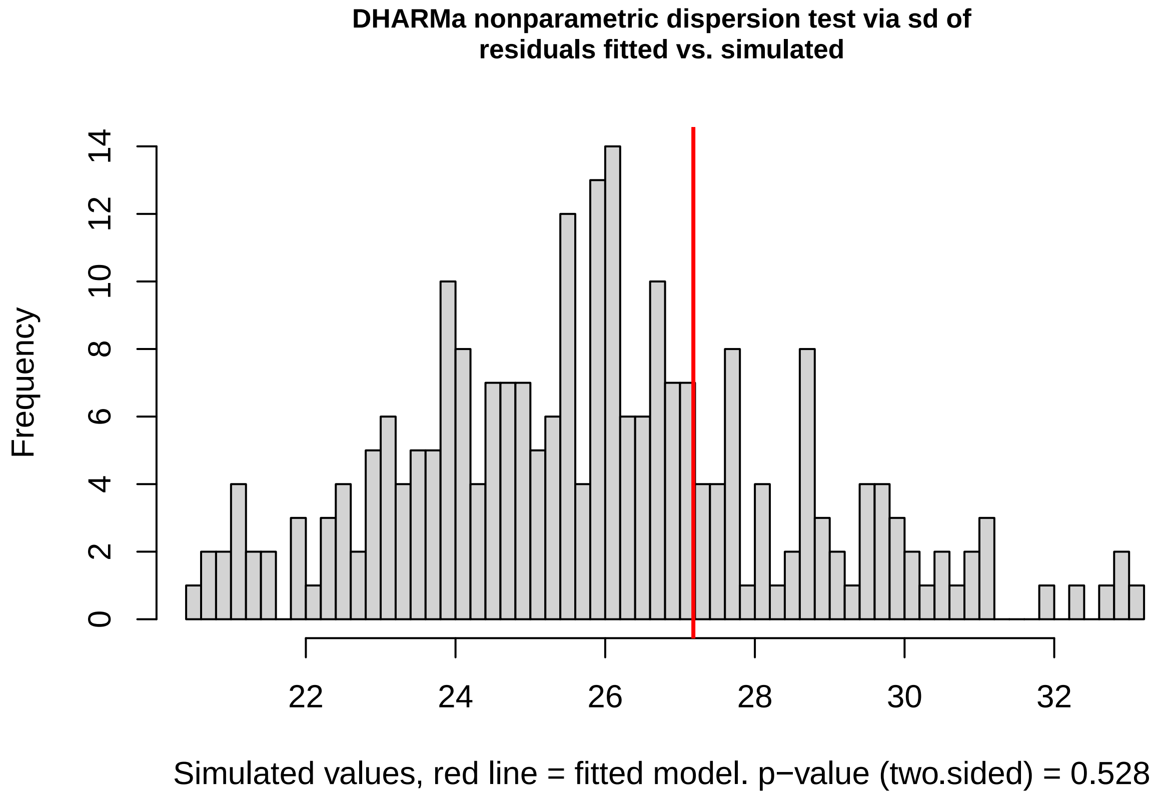


```
##
##  DHARMa nonparametric dispersion test via sd of residuals fitted vs.
##  simulated
##
## data:  simulationOutput
## ratioObsSim = 1.0496, p-value = 0.528
## alternative hypothesis: two.sided
```

```
##plot night & bat on IPI, no effect
plot_model(IPI_mod2, type="pred", terms=c("Night", "Bat"), pred.type="re",
           transform="exp", ci.lvl=0.95, show.data=TRUE, dot.size=1.2, colors = "system", line.size=1.2
  labs(y="Avg IPI (ms)", caption="IPI does not change across experimental night for all bats")+
  scale_x_continuous(breaks=c(1, 4, 7, 10), labels=scales::number_format(accuracy=1))+
  theme(axis.title.x=element_blank())
```

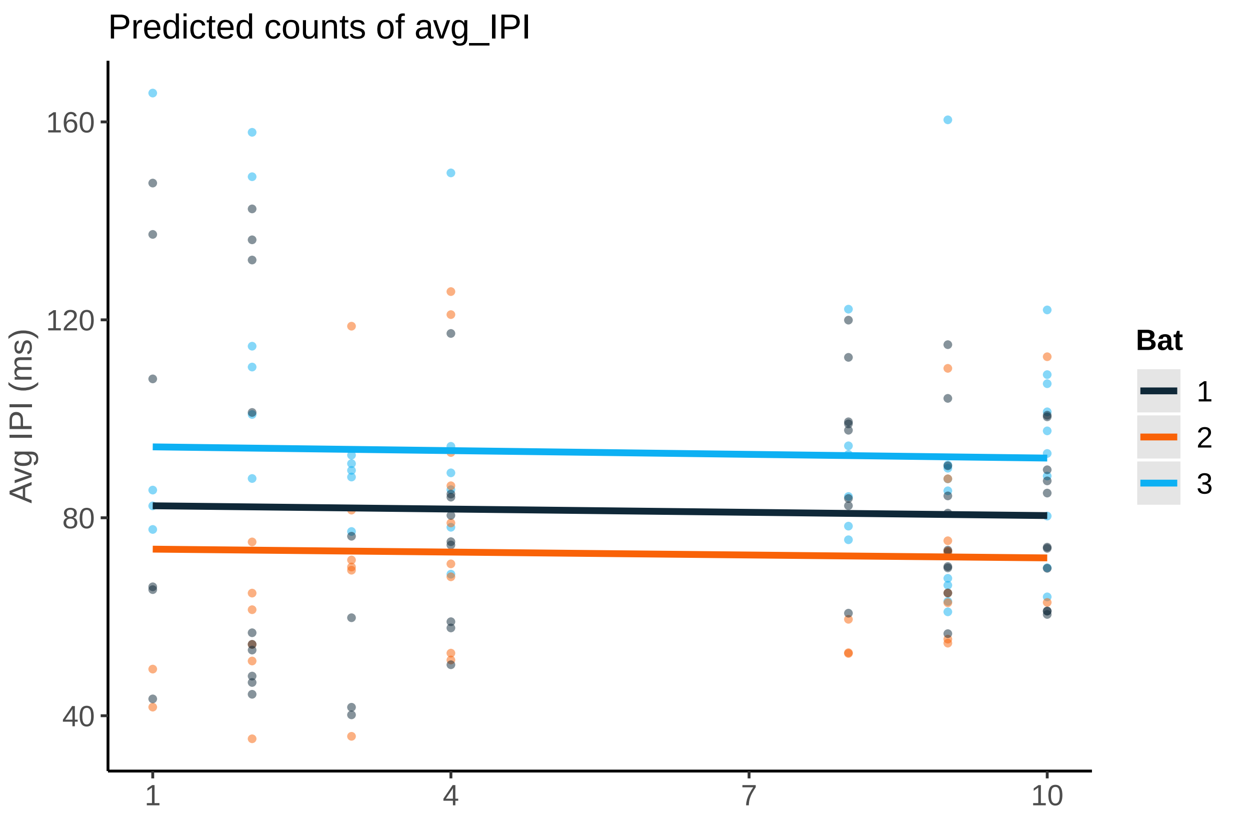

IPI does not change across experimental night for all bats

```r
###Model testing the effect of Treatment on Avg IPI###

IPI_mod3<-lmer(avg_IPI~Treatment+(1|Bat), data=acous)

summary(IPI_mod3)
```

```
## Linear mixed model fit by REML ['lmerMod']
## Formula: avg_IPI ~ Treatment + (1 | Bat)
##    Data: acous
##
## REML criterion at convergence: 1295.8
##
## Scaled residuals:
##     Min      1Q  Median      3Q     Max
## -1.9327 -0.6778 -0.1792  0.3582  2.7593
##
## Random effects:
##  Groups   Name        Variance Std.Dev.
##  Bat      (Intercept)  71.11    8.433
##  Residual             586.66   24.221
## Number of obs: 142, groups:  Bat, 3
##
## Fixed effects:
##                        Estimate Std. Error t value
## (Intercept)              94.850      5.935  15.983
## TreatmentOverlapping    -18.593      5.255  -3.538
```

```
## TreatmentNon-overlapping  -20.308      4.816  -4.217
##
## Correlation of Fixed Effects:
##             (Intr) TrtmnO
## TrtmntOvrlp -0.373
## TrtmntNn-vr -0.402  0.461
```

```r
confint(IPI_mod3)
```

```
##                                2.5 %     97.5 %
## .sig01                      1.226643  21.817648
## .sigma                     21.478152  27.185755
## (Intercept)                81.908427 107.283074
## TreatmentOverlapping      -29.445383  -8.572017
## TreatmentNon-overlapping -30.058425 -11.069862
```

```r
#checking model fit
check_IPI_mod3<-simulateResiduals(fittedModel = IPI_mod3, n=1000, plot=T)
```

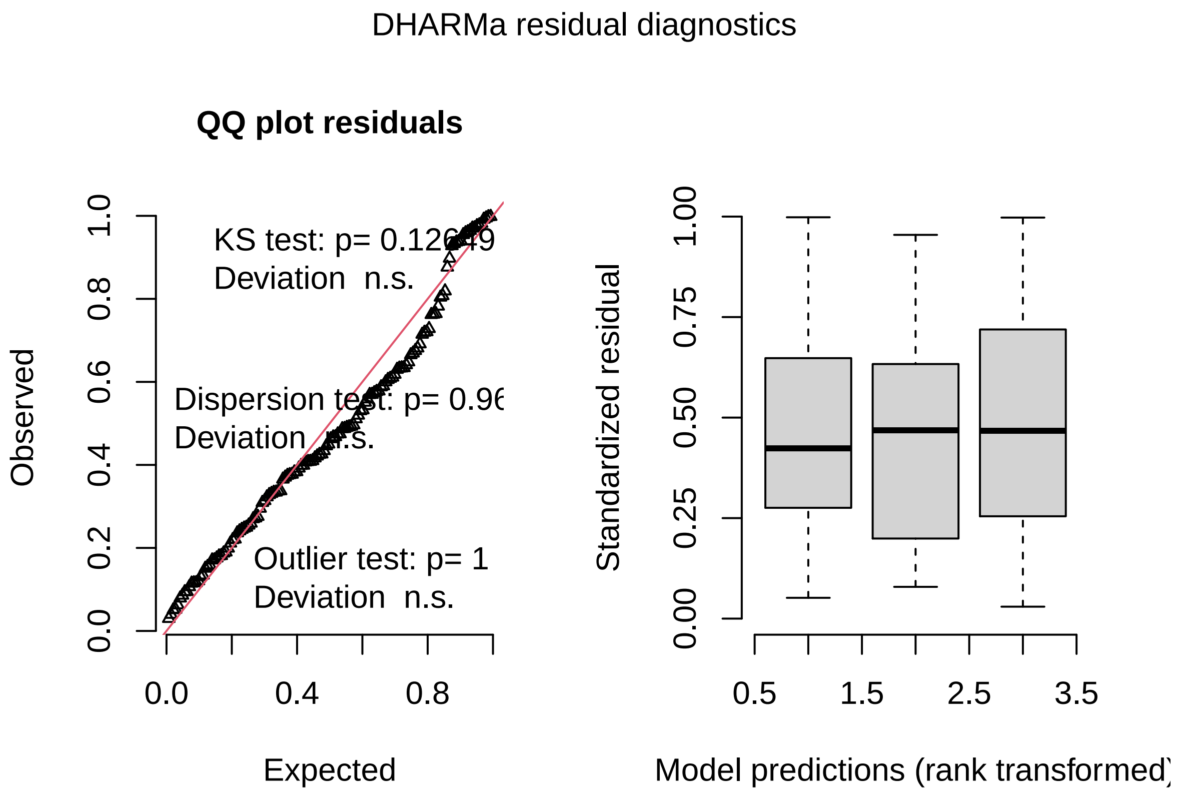


```r
testDispersion(IPI_mod3)
```

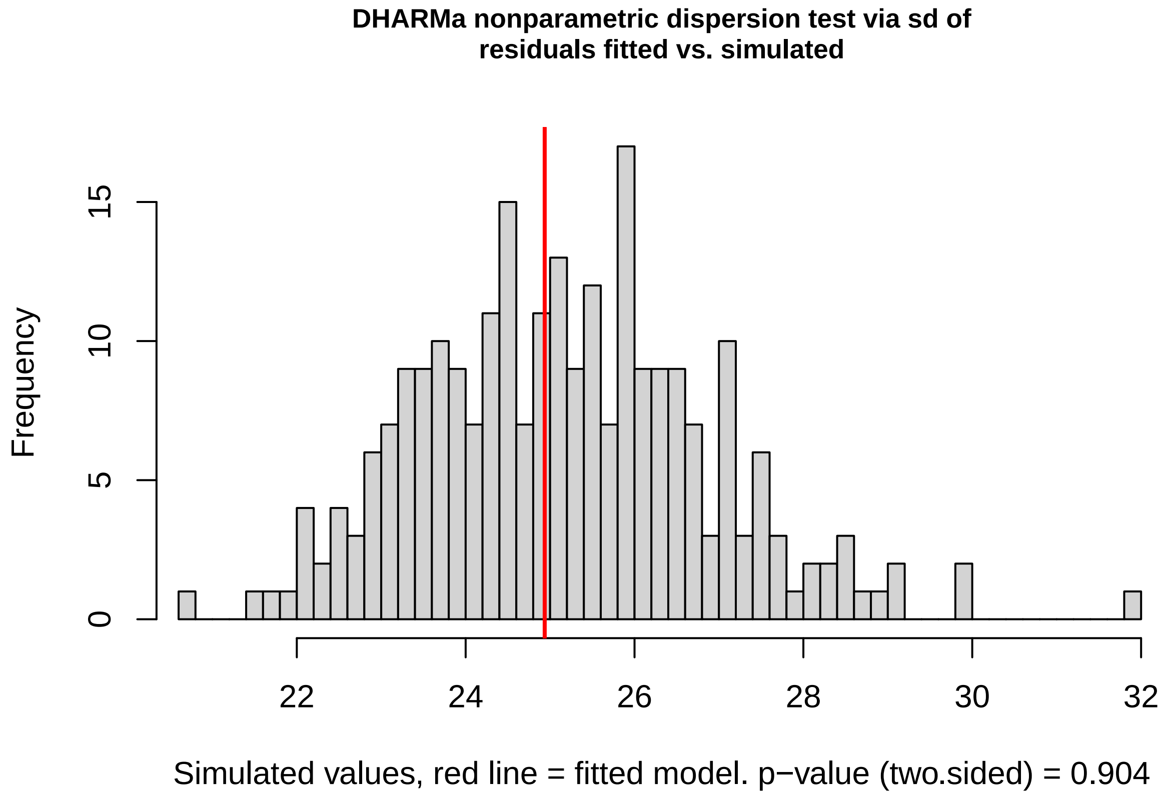


```
##
##  DHARMa nonparametric dispersion test via sd of residuals fitted vs.
##  simulated
##
## data:  simulationOutput
## ratioObsSim = 0.9907, p-value = 0.904
## alternative hypothesis: two.sided
```

```
###DOMINANT FREQUENCY####

###Model testing the effect of prey reward on trial outcome###

dfreq_mod1<-lmer(avg_dfreq~Treatment*MW_absent+(1|Bat), data=acous)
summary(dfreq_mod1)
```

```
## Linear mixed model fit by REML ['lmerMod']
## Formula: avg_dfreq ~ Treatment * MW_absent + (1 | Bat)
##    Data: acous
##
## REML criterion at convergence: 773.3
##
## Scaled residuals:
##     Min      1Q  Median      3Q     Max
## -4.8104 -0.5269  0.0470  0.6491  2.5979
##
## Random effects:
##  Groups   Name        Variance Std.Dev.
```

```
##  Bat      (Intercept)  0.1461  0.3822
##  Residual             15.2610  3.9065
## Number of obs: 142, groups:  Bat, 3
##
## Fixed effects:
##                                     Estimate Std. Error t value
## (Intercept)                          78.9099     0.5894 133.876
## TreatmentOverlapping                  0.6435     0.8921   0.721
## TreatmentNon-overlapping             -1.3208     0.8256  -1.600
## MW_absent1                            2.4285     2.8156   0.862
## TreatmentOverlapping:MW_absent1      -3.5844     3.2569  -1.101
## TreatmentNon-overlapping:MW_absent1  -1.2182     3.1356  -0.389
##
## Correlation of Fixed Effects:
##             (Intr) TrtmnO TrtmN- MW_bs1 TO:MW_
## TrtmntOvrlp -0.569
## TrtmntNn-vr -0.615  0.408
## MW_absent1  -0.174  0.115  0.124
## TrtmnO:MW_1  0.149 -0.268 -0.106 -0.865
## TrtmN-:MW_1  0.159 -0.105 -0.261 -0.898  0.776
```

```
check_mod_dfreq1 <- simulateResiduals(fittedModel = dfreq_mod1,n=1000, plot=T)
```

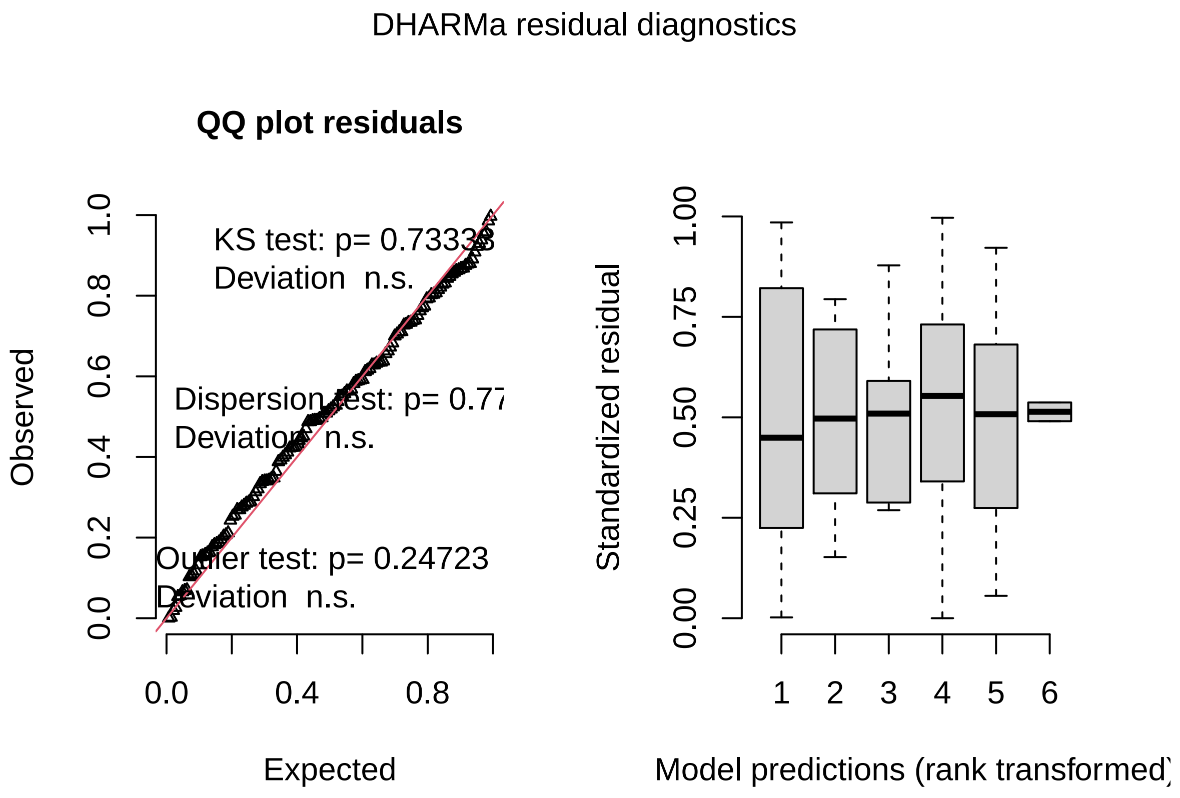


```
testDispersion(dfreq_mod1)
```

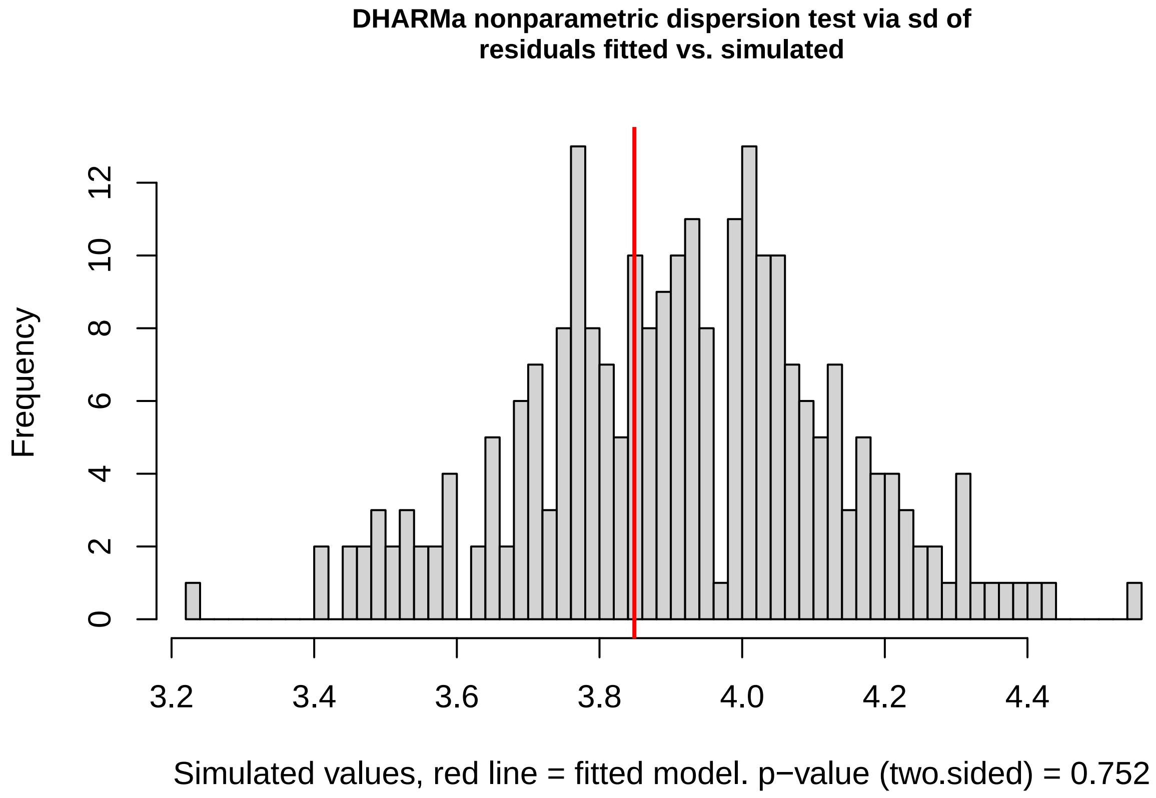


```
##
##  DHARMa nonparametric dispersion test via sd of residuals fitted vs.
##  simulated
##
## data:  simulationOutput
## ratioObsSim = 0.98315, p-value = 0.752
## alternative hypothesis: two.sided
```

```
#Interaction plot for prey reward on dominan freq.
plot_model(dfreq_mod1, type="int", colors="ipsum")+
labs(y="Avg dominant frequency (kHz)",caption="Interaction of treatment and presence of prey reward on 
                                                                                                     There is 
     title=element_blank())+theme(axis.title.x=element_blank())
```

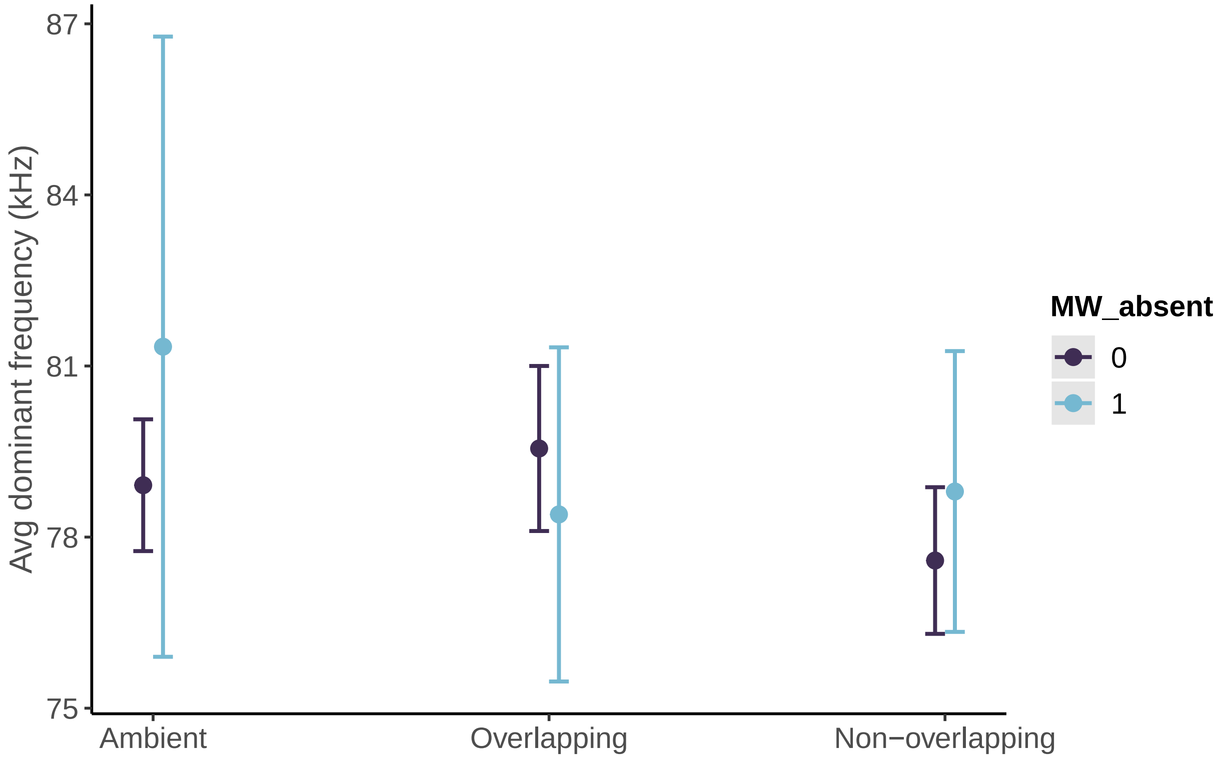

Interaction of treatment and presence of prey reward on the board.
There is no difference of dominant frequency with prey on or off the board.

```
###Model testing the effect of Treatment on dom. frequency###

dfreq_mod2<-lmer(avg_dfreq~Treatment+(1|Bat), data=acous)

summary(dfreq_mod2)
```

```
## Linear mixed model fit by REML ['lmerMod']
## Formula: avg_dfreq ~ Treatment + (1 | Bat)
##    Data: acous
##
## REML criterion at convergence: 784.5
##
## Scaled residuals:
##     Min      1Q  Median      3Q     Max
## -4.8465 -0.5196  0.1134  0.6153  2.5723
##
## Random effects:
##  Groups   Name        Variance Std.Dev.
##  Bat      (Intercept)  0.1996  0.4467
##  Residual             15.1273  3.8894
## Number of obs: 142, groups:  Bat, 3
##
## Fixed effects:
##                          Estimate Std. Error t value
## (Intercept)               78.9824     0.5946 132.833
## TreatmentOverlapping       0.3713     0.8321   0.446
```

```
## TreatmentNon-overlapping  -1.1507     0.7674  -1.499
##
## Correlation of Fixed Effects:
##             (Intr) TrtmnO
## TrtmntOvrlp -0.582
## TrtmntNn-vr -0.628  0.452
```

```
confint(dfreq_mod2)
```

```
##                                2.5 %     97.5 %
## .sig01                      0.000000  1.6440113
## .sigma                      3.450215  4.3612406
## (Intercept)                77.775086 80.1058443
## TreatmentOverlapping       -1.368485  1.9397355
## TreatmentNon-overlapping   -2.710890  0.3046862
```

```
check_mod_dfreq2 <- simulateResiduals(fittedModel = dfreq_mod2,n=1000, plot=T)
```

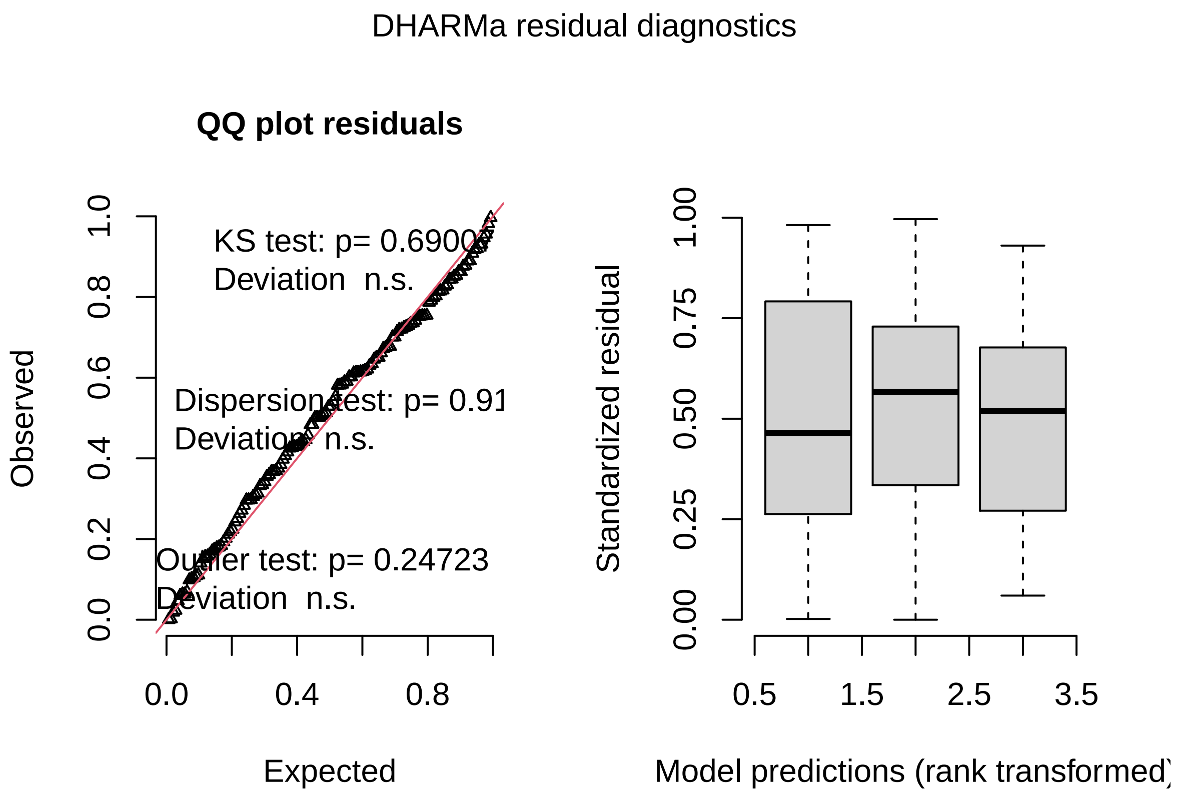


```
testDispersion(dfreq_mod2)
```

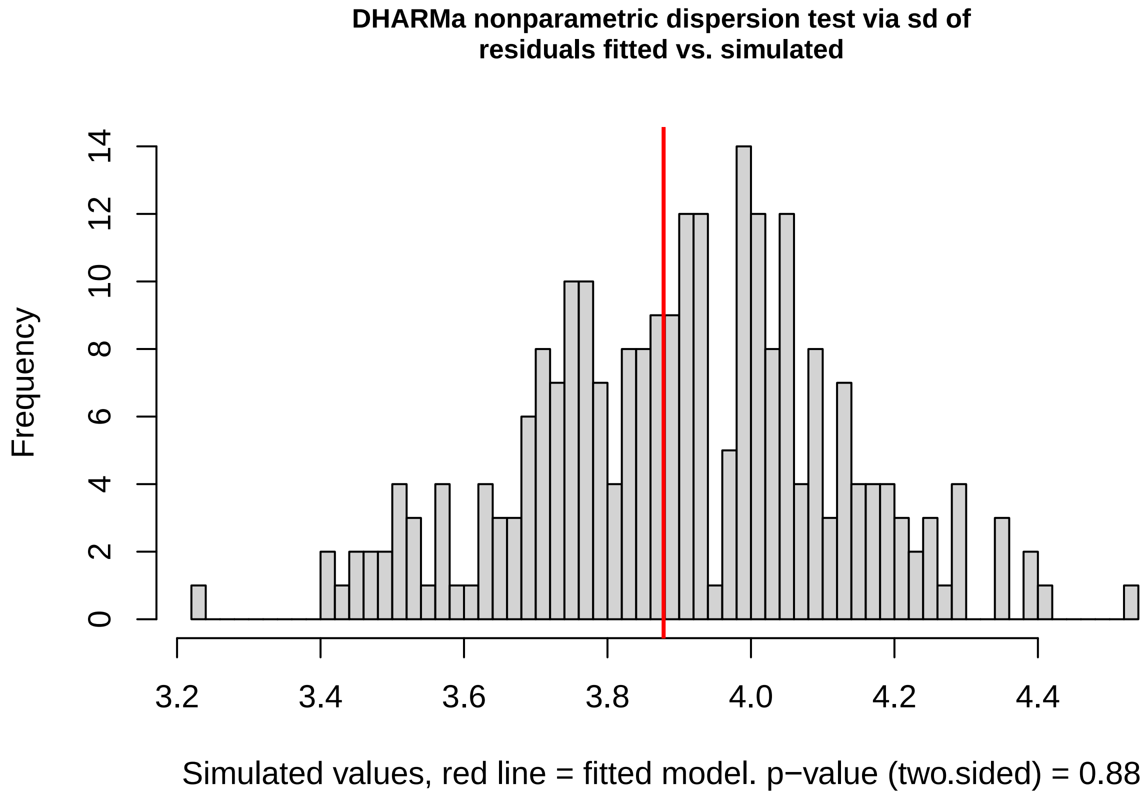


```
##
##  DHARMa nonparametric dispersion test via sd of residuals fitted vs.
##  simulated
##
## data:  simulationOutput
## ratioObsSim = 0.99369, p-value = 0.88
## alternative hypothesis: two.sided
```

```
#Plot of the effect of Treatment on dom. frequency
ggplot(acous, aes(x=Treatment, y=avg_dfreq))+geom_boxplot()+geom_jitter(color="black", size=0.4, alpha=
```

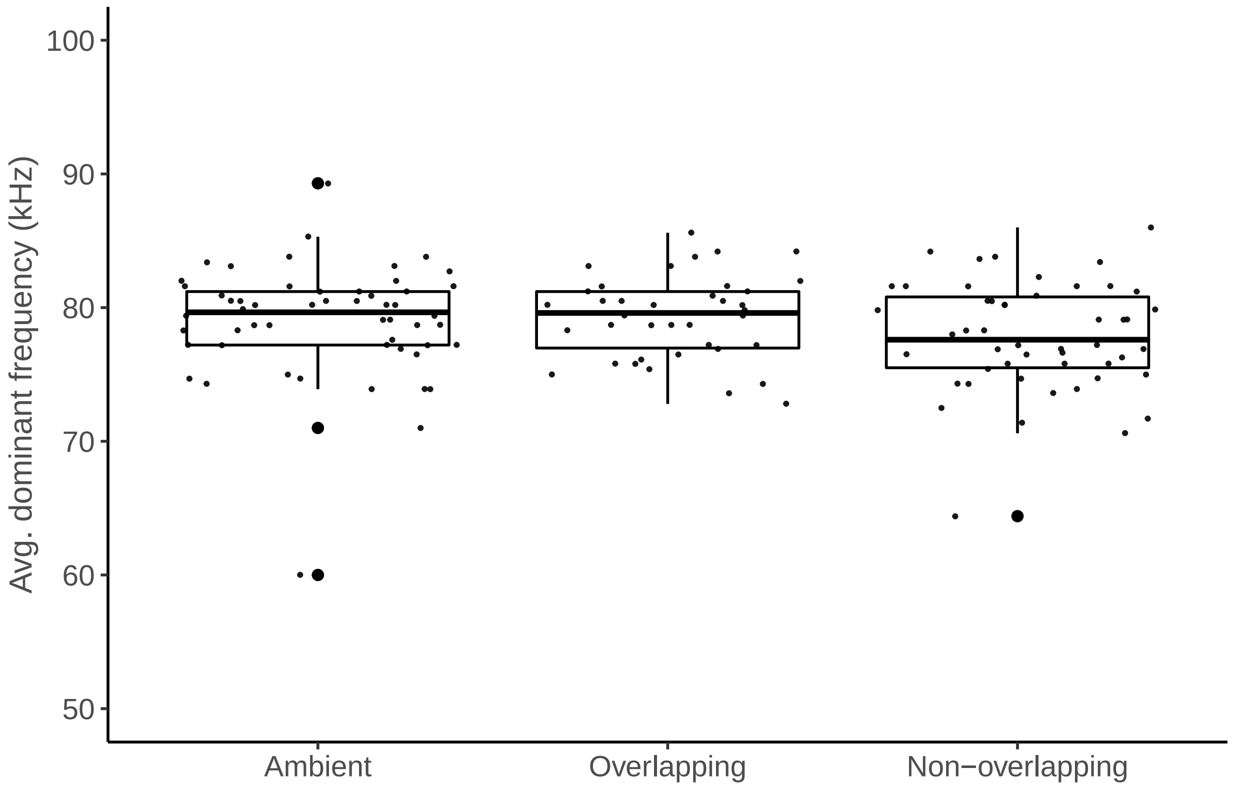

Treatment type does not affect dominant frequency of bat sonar

```
##Attempt plot##

#getting Attempt probabilities
dat1%>%
  mutate(pred_prob=predict(Attempt_mod3, type="response"))->pred_dat

colors1<-c("Ambient"="gray58", "Overlapping"="midnightblue", "Non-overlapping"="gold1")
Attempt_plot<-ggplot(pred_dat, aes(x=Night, y=Attempt, color=Treatment))+stat_smooth(method="glm", aes(


##Success plot##
set_theme(
  base=theme_blank(), axis.linecolor = "black", axis.ticksize.y = 0.2)

me<-ggpredict(success_mod3, c("Treatment"))
Success_plot<-ggplot(me, aes(x=x, y=predicted, color=x))+geom_point(cex=2)+
  geom_errorbar(aes(ymin=conf.low, ymax=conf.high), width=0.15)+scale_color_manual(values=c("Ambient"="



##Search Time plot##
set_theme(
  base=theme_blank(), axis.linecolor = "black", axis.ticksize.y = 0.2)

comparisons<-list(c("Ambient","Overlapping"),c("Ambient","Non-overlapping"),c("Non-overlapping", "Overla
ST_plot<-ggplot(dat2, aes(x=Treatment,y=Search_time, color=Treatment))+
```

```
  geom_boxplot()+scale_color_manual(values=c("gray58","midnightblue","gold1"))+
  geom_jitter(color="gray40", size=0.5, alpha=0.9) +
  ggpubr::stat_compare_means(comparisons=comparisons, label.y=c(50, 55, 61),label="p.signif")+
  theme(legend.position="none", axis.title.x=element_blank(), axis.text.x=element_text(size=15), axis.t



##Avg IPI plot##
set_theme(
  base=theme_blank(), axis.linecolor = "black", axis.ticksize.y = 0.2)

IPI_plot<-ggplot(acous, aes(x=Treatment,y=avg_IPI, color=Treatment))+
  geom_boxplot()+scale_color_manual(values=c("gray58","midnightblue","gold1"))+
  geom_jitter(color="gray40", size=0.5, alpha=0.9)+
  stat_compare_means(comparisons=comparisons, label.y=c(180, 190, 205),label="p.signif")+
  theme(legend.position="none", axis.title.x=element_blank(), axis.text.x=element_text(size=15), axis.t


#Making combined plot
#library("useful")
#library(grid)
#png(filename="Fig2_draftb.png", width=500, height=1000, units="px", 12)
#grid.newpage()
#pushViewport(viewport(layout = grid.layout(3,1)))
#print(Success_plot, vp = vplayout(1,1))
#print(ST_plot, vp = vplayout(2,1))
#print(IPI_plot, vp = vplayout(3,1))

#dev.off()
```
